# An Outlook on Platinum-Based Active Ingredients for Dermatologic and Skincare Applications

**DOI:** 10.3390/nano14151303

**Published:** 2024-08-02

**Authors:** Shining Li, Yizhou Liu, Ying Wu, Lu Ren, Yongjie Lu, Shuji Yamaguchi, Qipeng Lu, Chuangang Hu, Dongcui Li, Naisheng Jiang

**Affiliations:** 1 Key Laboratory of Advanced Materials and Devices for Post-Moore Chips, Ministry of Education, School of Materials Science and Engineering, University of Science and Technology Beijing, Beijing 100083, China; 2 Hua An Tang Biotech Group Co., Ltd., Guangzhou 511434, China; 3 CLINICA BellaForma, Tokyo 107-0052, Japan; 4 State Key Laboratory of Organic–Inorganic Composites, College of Chemical Engineering, Beijing University of Chemical Technology, Beijing 100029, China

**Keywords:** platinum nanoparticles, platinum-based active ingredients, antioxidant properties, anti-inflammatory, dermatology, skincare applications

## Abstract

Platinum-based materials exhibit a broad spectrum of biological activities, including antioxidant, anti-inflammatory, antimicrobial, and pro-collagen synthesis properties, making them particularly useful for various biomedical applications. This review summarizes the biological effects and therapeutic potential of platinum-based active ingredients in dermatological and skincare applications. We discuss their synthesis methods and their antioxidant, anti-inflammatory, antimicrobial, and collagen synthesis properties, which play essential roles in treating skin conditions including psoriasis and acne, as well as enhancing skin aesthetics in anti-aging products. Safety and sustainability concerns, including the need for green synthesis and comprehensive toxicological assessments to ensure safe topical applications, are also discussed. By providing an up-to-date overview of current research, we aim to highlight both the potential and the current challenges of platinum-based active ingredients in advancing dermatology and skincare solutions.

## 1. Introduction

Platinum (Pt), a noble metal with an atomic number of 78, has been valued for centuries due to its outstanding resistance to corrosion and oxidation, lustrous appearance, and remarkable ductility and malleability. Historically, its aesthetic appeal and resilience to wear and tarnishing have made it a preferred choice in fine jewelry [1]. Its high melting point and catalytic properties have also facilitated diverse industrial applications, ranging from electrical contacts and electrodes to the manufacturing of silicone and benzene [2,3]. With advancements in modern chemistry and the development of nanotechnology, platinum has been engineered into various nanoscale forms, such as platinum nanoparticles (PtNPs), nanorods, nanowires, and nanoclusters. Each form exhibits unique physicochemical properties that are applied across a spectrum of fields, from electronics and catalysis to biomedicine [4,5]. For example, platinum nanowires (PtNWs) are utilized as electrodes in fuel cells and sensors due to their excellent conductivity and large surface area, which allow for more effective energy conversion and more sensitive detection capabilities [6,7,8,9]. In industrial catalysis, platinum-based (Pt-based) materials are crucial for automotive catalytic converters, where they can accelerate the breakdown of noxious gases into less harmful substances, thereby reducing vehicle emissions [10]. In biomedicine, PtNPs are explored for their potential in targeted therapeutic interventions, particularly in oncology. Their ability to catalyze the production of reactive oxygen species (ROS) is employed to selectively induce apoptosis in tumor tissues, thereby minimizing damage to surrounding healthy cells [11]. This selective cytotoxicity is further enhanced by functionalizing the surface of PtNPs to improve interactions with specific biological molecules, thus improving their efficacy in drug delivery systems and biosensing applications [12,13]. Furthermore, platinum is also used to produce cytostatic drugs such as carboplatin, cisplatin, and oxaliplatin, which are commonly used to treat various types of cancer [14,15].

Beyond their established roles in catalysis and biomedicine, Pt-based materials have received increasing attention for their potential in dermatology and skincare. These materials, known as Pt-based active ingredients, utilize the unique properties of platinum to enhance the efficacy of skin treatments and personal care formulations [16]. They are also specifically designed and engineered to maximize biocompatibility and stability, ensuring their safety and effectiveness for long-term use [17]. Recent studies have highlighted the role of Pt-based active ingredients in regulating oxidative stress and mitigating inflammatory responses [18,19]. For example, studies by Kajita et al. [20] demonstrated the efficacy of PtNPs in scavenging reactive oxygen species (ROS), while Yoshihisa et al. [21] showed that PtNPs are capable of treating UV-induced epidermal inflammation. It was also found that the inherent antioxidant properties of PtNPs can help to neutralize harmful free radicals, thereby slowing the skin aging process [22]. Their anti-inflammatory properties can also help to reduce skin inflammation and promote overall skin health [23]. The excellent biocompatibility of PtNPs can reduce the risk of skin allergies and adverse reactions, making them suitable for sensitive skin types [24]. These findings suggest that incorporating Pt-based active ingredients in cosmetic formulations not only enhances product efficacy but also meets the growing consumer demand for dermatological treatment and premium skincare solutions.

In this review, we discuss the potential role of Pt-based active ingredients in dermatology and skincare applications. In the next section, we focus on the synthesis methods and surface functionalization of Pt-based active ingredients, emphasizing their physicochemical properties and the techniques used to enhance their biological activity. Section 3 focuses on the biological effects of these Pt-based materials, highlighting their antioxidant, anti-inflammatory, and antibacterial properties, as well as their roles in promoting collagen synthesis and influencing melanogenesis. In Section 4, we delve into practical applications, evaluating how Pt-based active ingredients can enhance cosmetic skincare products and treat inflammatory skin conditions. In Section 5, we discuss the safety and sustainability of Pt-based materials for dermatological applications. Finally, we summarize the main points discussed throughout this review and provide perspectives on the future potential of Pt-based active ingredients in dermatology and skincare applications. It should be noted that in this review, the term “Pt-based material” refers to all materials containing platinum, while “platinum nanoparticles (PtNPs)” specifically refers to platinum in nanoparticle form. In addition, “Pt-based active ingredients” refers to substances containing platinum that are specifically used for their functional roles in formulations intended for dermatological treatments or skincare products. These ingredients should utilize the unique properties of platinum to enhance the effectiveness and target specific therapeutic or cosmetic outcomes.

## 2. Synthesis and Functionalization of Platinum-Based Active Ingredients

Platinum can be synthesized into various particle forms, ranging from nanoscale to mesoscale, displaying diverse crystalline faces, morphologies, and physicochemical properties [25]. Among these forms, PtNPs with nanometer dimensions exhibit distinctive characteristics that deviate from their bulk counterparts, such as an increased surface area-to-volume ratio, which significantly enhances their catalytic activity [26]. This makes them highly effective for applications extending from chemical catalysis to medical therapy [27,28]. PtNPs can be engineered into various geometric shapes, including cubes, tetrahedrons, octahedrons, cuboctahedrons, decahedrons, and icosahedrons [29,30], and predominantly display low-index facets such as {100} and {111}, which are thermodynamically stable and have densely packed atomic configurations [31,32]. On the other hand, high-index facets such as {310}, {311}, and {331}, although less thermodynamically stable, can provide a higher density of catalytically active sites compared to those conventional low-index facets, thereby enhancing their catalytic performance [33,34]. Despite their catalytic activity, however, PtNPs have raised concerns in biomedical applications regarding their potential toxicity, which is not fully understood yet [35]. On the other hand, larger platinum particles, typically in the submicron range, exhibit reduced surface reactivity and slower dissolution rates. These features enhance their biocompatibility and reduce toxicity concerns in biomedical applications, though they may result in diminished catalytic efficiency [36,37].

**Figure 1:**
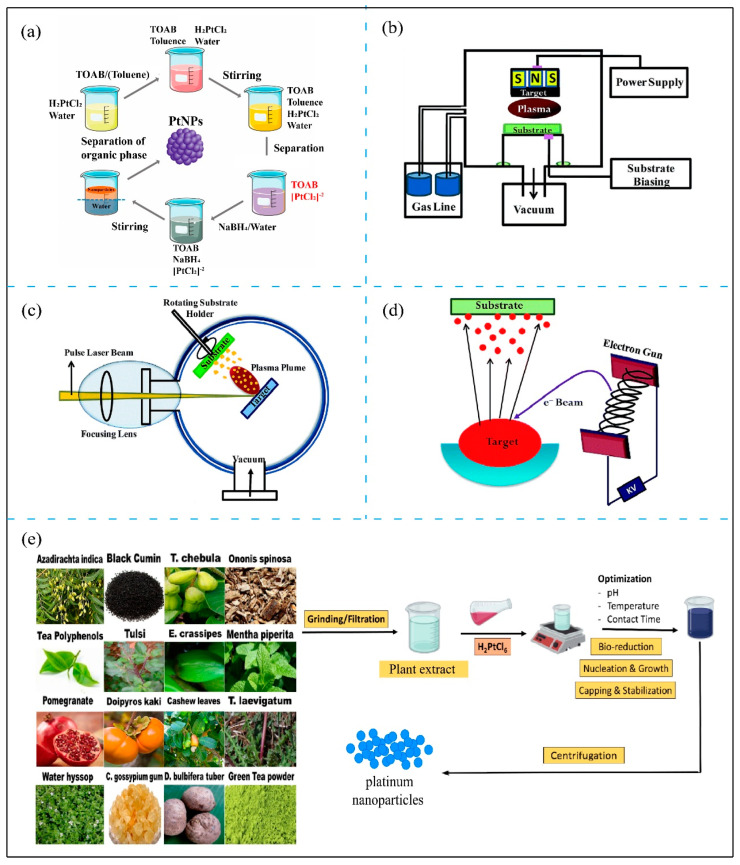
(**a**) Schematic illustration of the chemical reduction method for synthesizing PtNPs. Reproduced under the terms of the Creative Commons CC-BY license [62]. Copyright 2019, the authors, published by MDPI. Note: TOAB refers to Tetra-n-octylammonium bromide. (**b**–**d**) Schematic illustration of various physical synthesis methods: (**b**) plasma sputtering, (**c**) electron beam evaporation, and (**d**) pulsed laser deposition. Reproduced with permission [63]. Copyright 2015, Royal Society of Chemistry. (**e**) Schematic diagram summarizing the plant species and optimization parameters for obtaining biocompatible PtNPs using “green reagents” (modified from ref. [62]). Reproduced under the terms of the Creative Commons CC-BY license [64]. Copyright 2020, the authors, published by MDPI.

A variety of synthetic methods have been developed to tailor the structure and properties of Pt-based materials for specific applications [38,39], including chemical synthesis, physical synthesis, and biosynthesis. Among these methods, chemical synthesis usually provides the greatest control over the size, shape, and surface characteristics of synthesized particles [40]. The wet chemical reduction method is one of the most widely employed chemical synthesis techniques due to its effectiveness in controlling nanoparticle properties (Figure 1a) [41]. This method generally involves reducing platinum precursors, such as chloroplatinic acid, with suitable agents such as sodium borohydride, hydrogen, or formic acid, often in aqueous solutions. Parameters such as the reagent ratios, reaction temperature, and pH are crucial in tailoring the size, shape, and surface properties of the resulting PtNPs [42], which can affect the catalytic activity and stability of the nanoparticles. To enhance the catalytic performance, substantial efforts have been made to produce PtNPs with well-defined shapes through wet chemical reduction [43,44,45,46,47,48]. Various shape-directing agents have been employed, such as polymers and surfactants, to facilitate the asymmetric growth of nanoparticles, which is crucial for applications that require unique surface properties. The incorporation of organic or inorganic ligands can further refine the size and shape during synthesis. Stabilizers or capping agents such as polyvinyl alcohol, citrate, or polyvinylpyrrolidone can be added to the reaction mixture to prevent unwanted aggregation and ensure good dispersion of nanoparticles over time by forming a steric barrier. Moreover, innovative multiphase synthetic setups, such as introducing reducing agents in the gas phase, have been explored to achieve finer control over reaction parameters [49,50]. While these methods enhance control over nanoparticle synthesis, the extensive use of surfactants, capping agents, and organic solvents can potentially introduce toxicity or environmental concerns. Consequently, researchers have developed more environmentally friendly synthetic methods, employing microwave heating and glycerol both as reducing agents and solvents [51]. Green reagents, such as ascorbic acid and sodium citrate, have also been used to exert stringent control over the physicochemical properties of PtNPs while ensuring their biocompatibility [52]. Nevertheless, it should be noted that the presence of toxic chemicals and solvents in chemical synthesis can pose significant health risks, including skin irritation or allergic reactions. Thus, it is crucial to thoroughly remove these toxic contaminants and residual substances from the final products, especially in skincare applications where prolonged skin contact is expected, to ensure user safety and product efficacy.

On the other hand, physical synthesis methods, such as laser ablation [53,54], aerosol-assisted deposition [55], electron-beam-induced reduction [56], and flame synthesis [57,58], among many others, offer alternative routes to produce PtNPs without using toxic reagents and solvents. Examples of these synthesis methods are shown in Figure 1b–d. Laser ablation, for example, uses a high-energy laser to evaporate platinum from a solid source into a liquid medium, where the nanoparticles condense [59]. This method can be operated in either continuous or pulsed modes, allowing one to adjust pulse intensity, temperature, and ambient gas pressure to finely tune the properties of the nanoparticles. Similarly, aerosol-assisted deposition involves generating a platinum aerosol that is subsequently deposited to form nanoparticles [55], whereas the electron-beam-induced reduction method employs electron beams to reduce platinum ions to nanoparticles under suitable conditions [60]. Additionally, flame synthesis offers another approach by using a combustion flame to directly synthesize PtNPs from the gas phase [61]. Although these methods can produce PtNPs with high purity, seemingly ideal for dermatology and other biomedical applications, they often have low yields and require sophisticated and energy-intensive equipment, which limits their scalability and cost-effectiveness for commercial skincare products. In addition, challenges in precisely controlling the size, shape, and surface properties of the nanoparticles also hinder the practical use of these methods.

**Figure 2:**
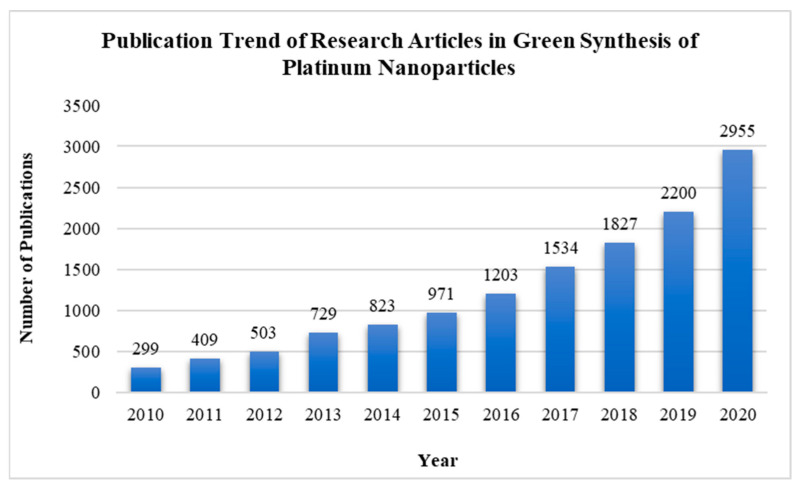
Publication trend regarding the green synthesis of platinum nanoparticles (PtNPs) (data obtained from ref. [71]). Reproduced with permission [73]. Copyright 2021, Elsevier.

Biosynthesis or biomolecule-assisted synthesis, which utilizes biological agents such as microorganisms, plant extracts (Figure 1e), or enzymes [62,65], offers an environmentally sustainable and green approach to producing PtNPs. These biological agents naturally reduce platinum ions to elemental platinum under ambient conditions. For example, the use of cyanobacteria, plant extracts, and even aqueous honey solutions in biosynthesis has been found to produce monodisperse and stable PtNPs [66,67,68]. Sulphate-reducing bacteria and plant extracts have also demonstrated their ability to reduce Pt (IV) into PtNPs [69,70]. These biological agents can also serve as natural capping agents to inherently stabilize the nanoparticles, which avoids the need for external stabilizers or toxic chemicals. The shift toward biosynthesized Pt-based materials, favored for their environmental benefits and non-toxicity, reflects a growing trend in research focused on sustainable nanoparticle production methods [71] (Figure 2). Apparently, biosynthesized platinum is highly suitable for topical applications due to its gentle synthesis process and natural compatibility with human skin, making it ideal for dermatology and skincare applications. The concept of the green synthesis approach also aligns with increasing consumer demand for natural and organic skincare products. Despite these advantages, a major challenge in biosynthesis is the difficulty in achieving consistent nanoparticle size and morphology. Variations in biological material and synthesis conditions often result in batch-to-batch inconsistencies, creating obstacles for the standardization crucial for commercial scalability. Furthermore, the presence of biological contaminants such as endotoxins [72], which are costly and challenging to remove, may hinder the large-scale commercial use of biosynthesized platinum. Ongoing research is focused on overcoming these challenges to improve the reliability and reduce the ecological footprint of biosynthesized platinum, thereby enhancing its appeal for the skincare industry and other biomedical applications.

As summarized in Table 1, PtNPs synthesized through various methods can exhibit significant differences in size, shape, dispersive state, and other properties. Typically, the average size of these particles ranges from 1 to 100 nm, with the majority being smaller than 5 nm, and spherical shapes are the most commonly observed morphology. Note that for dermatological applications, biosynthetic methods are predominantly used due to their potential for biocompatibility [74]. Although synthesis methods may vary significantly across different studies, it is evident that each synthesis method should be carefully selected and optimized with suitable reducing agents, stabilizers, and synthesis conditions to produce PtNPs with desired properties.

**Figure 3:**
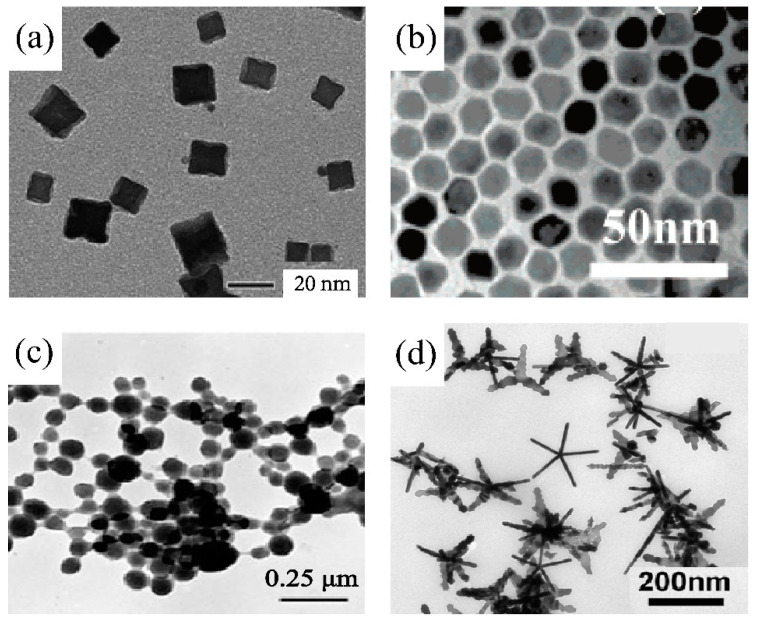
Representative morphologies of platinum nanoparticles (PtNPs). (**a**) TEM image of platinum concave nanocubes synthesized by continuously adding an aqueous NaBH_4_ solution and a mixture of K_2_PtCl_4_, KBr, and Na_2_H_2_P_2_O_7_ using two syringe pumps into deionized water held at 95 °C. Reproduced with permission [91]. Copyright 2011, John Wiley and Sons. (**b**) TEM image of platinum icosahedra with an edge length of 8.8 nm. Reproduced with permission [92]. Copyright 2007, American Chemical Society. (**c**) TEM image showing spherical platinum particles forming long bead-like chains. Reproduced under the terms of the Creative Commons CC-BY license [93]. Copyright 2021, the authors, published by Frontiers. (**d**) TEM image of star-shaped platinum. Reproduced with permission [94]. Copyright 2012, John Wiley and Sons.

As aforementioned, Pt-based materials synthesized through various methods exhibit distinct morphologies (Figure 3), and their functionalization is crucial for transforming them into active ingredients for dermatology and skincare. Common strategies such as polymer grafting or PEGylation, ligand attachment, and surface charge modification are designed to enhance their stability, biocompatibility, and overall effectiveness. For example, thiol-chemistry can stabilize PtNPs in both aqueous and organic media using alkane thiols or polar-group-bearing thiols as capping agents to prevent aggregation [85,86,87]. Additionally, modifying thiol ligands with various functional groups enables targeted interactions with specific biological sites [88]. Active agents containing thiol groups can be chemically tailored to interact with a variety of organic biological molecules, including polypeptides, polysaccharides, proteins, nucleotides, and antibodies [89], allowing for customized modifications as required. It is thus important to select agents with suitable molecular weights and chemical structures to ensure conjugation stability on PtNPs for cosmetic applications. Note that while these functionalization methods can improve stability and dispersion, they may also reduce the intrinsic catalytic activity of PtNPs by covering active sites or altering surface properties essential for catalysis [90]. Compatibility testing is advisable when incorporating functionalized Pt-based active ingredients into cosmetic formulations to ensure they do not adversely affect the stability and therapeutic efficacy of the final product.

## 3. Biological Effects of Pt-Based Active Ingredients

### 3.1. Antioxidant Properties

**Figure 4:**
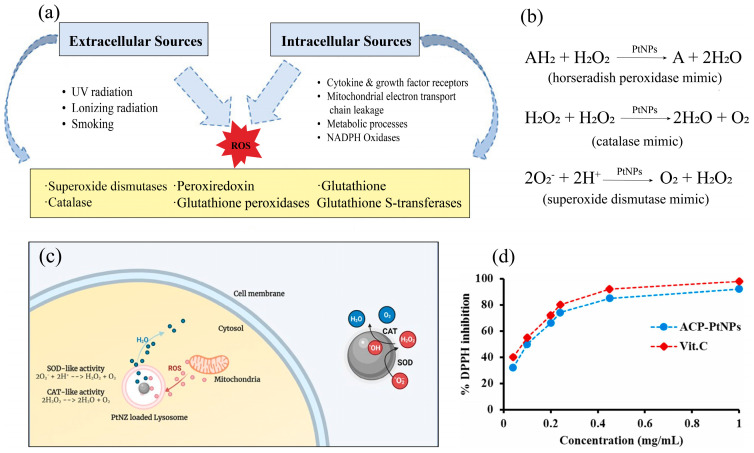
(**a**) Various sources of reactive oxygen species (ROS). (**b**) Schemes of the main antioxidant chemical reactions catalyzed by PtNPs as peroxidase (modified from ref. [82]). Reproduced with permission [96]. Copyright 2017, Royal Society of Chemistry. (**c**) Schematic illustration of the intracellular, platinum-based nanozyme (PtNZ) with catalase (CAT)-like and superoxide dismutase (SOD)-like activity. The PtNZ localized in the lysosome can act as ROS scavengers, preventing ROS from reaching abnormally high levels. Reproduced with permission [23]. Copyright 2023, Royal Society of Chemistry. (**d**) DPPH scavenging efficacies of vitamin C and PtNPs biosynthesized using acid phosphatase from *Rumex dentatus* seed extract (ACP-PtNPs, conc. = 0–1 µg/mL, DPPH conc. = 1 mM, vol. = 0.5 mL). Reproduced with permission [115]. Copyright 2022, Elsevier.

Pt-based active ingredients can exhibit excellent antioxidant properties that are useful in biomedical applications, such as dermatology and skincare [95,96]. For example, the catalytic activity of PtNPs can neutralize free radicals, thereby protecting skin tissues from oxidative damage and supporting natural defense systems against various stressors [97]. This catalytic property allows Pt-based active ingredients to engage in redox reactions that maintain intracellular redox balance, which is crucial for shielding cells from oxidative stress and mitigating skin damage from excessive reactive oxygen species (ROS) [98,99]. Note that ROS can be generated by different cellular sources within the cell and by different influences in the external environment (Figure 4a). While ROS are essential for regulating physiological functions [100], their excessive production can cause oxidative stress, potentially triggering inflammation and contributing to the development of various diseases such as cancer [101,102]. Studies have shown that PtNPs or Pt-based nanoenzymes can mimic the natural antioxidant enzymes, exhibiting enzymatic activities analogous to catalase (CAT), peroxidase (POD), superoxide dismutase (SOD), and glutathione peroxidase (GPx). They can catalyze the breakdown of hydrogen peroxide to water and oxygen and convert superoxide anions into less reactive species [103]. Thus, Pt-based nanoenzymes are able to decompose hydrogen peroxide into water by acting as a biological enzyme catalase catalyzing the reduction of hydrogen peroxide to water and molecular oxygen or as a horseradish peroxidase mimic facilitating the oxidation of reduced substrates (Figure 4b). They can also act as natural superoxide dismutase, catalyzing the cleavage of superoxide anions to molecular oxygen and hydrogen peroxide (Figure 4b). These Pt-based materials represent promising candidates for treating oxidative stress-related diseases by effectively managing cellular redox activities.

In a previous work, Kajita et al. [20] explored the antioxidant properties of dual-metal nanoparticles and compared citrate- and pectin-protected gold-platinum bimetallic nanoparticles (CP-Au/Pt) to nanoparticles solely composed of either metal. Bimetallic citrate- and pectin-protected gold-platinum bimetallic nanoparticles with a higher fraction of platinum (75% Pt and 25% Au) display enhanced antioxidant properties. These nanoparticles can effectively quench ROS, with those having higher platinum content showing higher quenching activity. This highlights the primary role of platinum in antioxidant processes. The citrate- and pectin-protected PtNPs (CP-Pt) exhibit the most potent quenching and antioxidant activity, with CP-Pt (average diameter of 4.7 ± 1.5 nm) quenching about 80% of hydrogen peroxide at 100 mM and about 60% of superoxide anions at 200 mM, and these results suggest their potential to mimic superoxide dismutase and catalase by quenching ROS (Figure 4c). Such capabilities indicate that PtNPs could be valuable in treatments for oxidative stress-related diseases and in anti-aging.

In the determination of the oxidation resistance of Pt-based materials, the DPPH assay [104,105], which utilizes the stabilizing radical 2,2-diphenyl-1-trinitrohydrazine (DPPH), is a widely used and effective method. In this assay, antioxidants are mixed with a DPPH solution and are tested for their ability to donate electrons or hydrogen atoms to neutralize the DPPH radical. This reaction reduces the light absorption by radicals, which can lead to a measurable color or absorbance change in the solution. The degree of absorbance change, captured by UV–visible spectrophotometry, quantifies the effectiveness of an antioxidant in reducing the concentration of free radicals [106]. The radical scavenging ability (i.e., percentage inhibition) of the antioxidant can thus be described by the following equation [107]:

(1) $$\mathrm{Radical}\;\mathrm{scavenging}\;\mathrm{ability}=\frac{\mathrm{optical}\;\mathrm{density}\;\mathrm{of}\;\mathrm{control}-\mathrm{optical}\;\mathrm{density}\;\mathrm{of}\;\mathrm{sample}}{\mathrm{optical}\;\mathrm{density}\;\mathrm{of}\;\mathrm{control}}\text{×}100\%$$

where optical density of control is the optical density (or absorbance) of the DPPH solution without the antioxidant, and optical density of sample is the optical density with the presence of the antioxidant in the solution. This metric allows one to assess the extent to which the antioxidant can mitigate oxidative damage by reducing ROS activity. It is worth noting that the terms “radical scavenging ability” and “radical inhibition” are often used interchangeably to describe how antioxidants reduce DPPH absorbance, both reflecting their effectiveness in neutralizing free radicals.

Studies have shown that the radical scavenging activity of Pt-based active ingredients can vary significantly, with few reaching or exceeding 90% inhibition to DPPH [108,109,110,111]. Moreover, their actual antioxidant properties can be affected by a variety of factors, including the synthesis method, the morphology, and the physicochemical properties of PtNPs, such as their shape, size, and surface properties, as well as the presence of other components or ingredients [112,113]. Notably, many studies have utilized biomolecule-assisted methods to synthesize these PtNPs due to safety considerations in potential biomedical and cosmetic applications. For example, Chen et al. [114] synthesized PtNPs using chlorogenic acid as both a reducing and stabilizing agent, which achieved a DPPH radical scavenging activity exceeding 95%. Subsequent tests after centrifugation showed that while the presence of chlorogenic acid enhances antioxidant activity, PtNPs themselves serve as the primary active components during the radical scavenging process, yielding a high inhibition value of above 90%. In another study, Rehman et al. [115] used acid phosphatase from *Rumex dentatus* seeds for synthesizing platinum nanoparticles utilizing the enzymatic activity of acid phosphatase to reduce platinum salts. These biosynthesized nanoparticles were found to scavenge DPPH radicals in a dose-dependent manner, reaching a peak DPPH scavenging rate of up to 88% at a concentration of 1 mg/mL (Figure 4d). In these experiments, vitamin C was used as a standard reference, highlighting the effectiveness of PtNPs in neutralizing ROS compared to traditional antioxidants. Similarly, Hosny et al. [116] synthesized PtNPs using the aqueous extract of *P. salicifolium* leaves via a green synthesis method. They found that increasing the concentration of PtNPs significantly enhanced their scavenging efficacy, with the DPPH clearance rate increased from 21% at 12.5 µg/mL to 90% at 50 µg/mL.

Table 2 summarizes the synthesis methods, particle morphologies, applied concentrations, and DPPH scavenging activities of various PtNPs along with other metal-based nanoparticles. While nanoparticles of the same metal may exhibit varying degrees of free radical scavenging abilities, the antioxidant performance of PtNPs often exceeds that of other metal nanoparticles. Antioxidant efficiencies that exceed 90% have been achieved by PtNPs formed via specific synthesis approaches. Such impressive radical-scavenging capability highlights the potential of PtNPs as an effective antioxidant agent for mitigating oxidative stress in skincare products. However, there is still a need for continued research to further clarify the key factors that enhance the antioxidant properties of Pt-based active ingredients.

### 3.2. Anti-Inflammatory Effects and Antibacterial Properties

**Figure 5:**
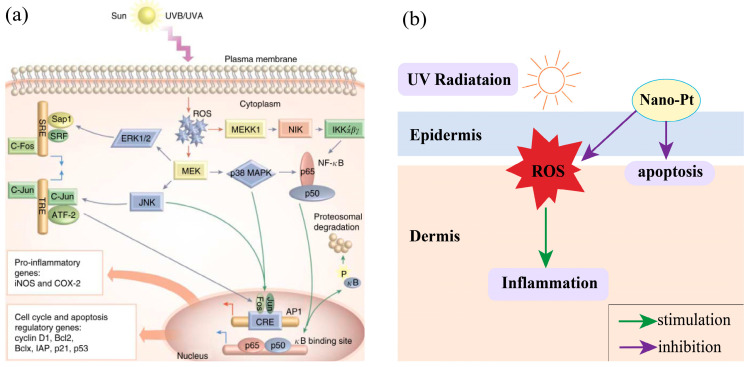
(**a**) Schematic illustration of reactive oxygen species (ROS)-mediated activation of various cell signaling pathways in the skin. Reproduced with permission [130]. Copyright 2006, Elsevier. (**b**) Schematic illustration of the protective effects of nano-sized platinum (nano-Pt) materials in UV-induced inflammation in the skin.

In addition to their antioxidant activity, Pt-based materials also exhibit anti-inflammatory and antimicrobial properties, making them beneficial for skincare and dermatological applications. The anti-inflammatory effects of PtNPs mainly rely on their ability to modulate oxidative stress within cells, a mechanism similar to their antioxidant properties. Excessive ROS, generated from cellular metabolic processes and exacerbated by environmental factors such as ultraviolet A or ultraviolet B (UVA/UVB) exposure, can disrupt cellular functions by damaging DNA, proteins, and lipids. This oxidative damage can activate several signaling pathways (Figure 5a), including the mitogen-activated protein kinases (MAPKs), such as extracellular signal-regulated kinase (ERK), c-Jun NH (2)-terminal kinase (JNK), and p38, as well as the transcription factor nuclear factor kappa-light-chain-enhancer of activated B cells (NF-κB). These pathways are crucial for regulating genes involved in inflammation, including inducible nitric oxide synthase (iNOS) and cyclooxygenase-2 (COX-2). Activation of these pathways can lead to increased production and release of inflammatory cytokines, exacerbating inflammatory responses [128,129,130]. By mitigating ROS, PtNPs can reduce the activation of these inflammatory pathways, thereby reducing the production of inflammatory mediators and alleviating symptoms associated with inflammatory skin conditions (Figure 5b).

Based on this anti-inflammatory mechanism, the anti-inflammatory properties of platinum have been incorporated into various formulations. A notable example is a mixture of palladium and platinum nanoparticles, which has been used in chronic disease treatments for over 60 years [131]. In vitro studies demonstrated that the palladium and platinum nanoparticle mixture has antioxidant activity against superoxide anion and hydroxyl radicals [132,133]. Reddy et al. [134] found that the palladium and platinum nanoparticle mixture, comprising 0.3 mg/mL (2.82 mM) of PdNPs and 0.2 mg/mL (1.03 mM) of PtNPs, could be percutaneously absorbed by cells, which plays a significant role in mitigating oxidative stress-induced skin lesions. Their studies suggest that the palladium and platinum nanoparticle mixture can reduce oxidative damage, which is known to downregulate type I collagen and hyaluronic acid synthesis, leading to skin atrophy and increased pro-inflammatory factors that exacerbate inflammation. This inflammation can further disrupt collagen homeostasis and ultimately cause skin-related problems. In addition, Tsuji et al. [135] suggested that the palladium and platinum nanoparticle mixture may protect keratinocytes from Interferon-γ (IFN-γ)-mediated cellular damage by upregulating NAD(P)H quinone dehydrogenase 1 (NQO1), an antioxidant enzyme in keratinocytes [136]. Inflammation reduction was also observed in human keratinocytes treated with the palladium and platinum nanoparticle mixture, which inhibited oxidative stress pathways by activating antioxidant mechanisms associated with Sod1 expression. These findings demonstrate that platinum in the mixture can effectively alleviate aging-related skin atrophy and inflammation by reducing oxidative damage.

**Figure 6:**
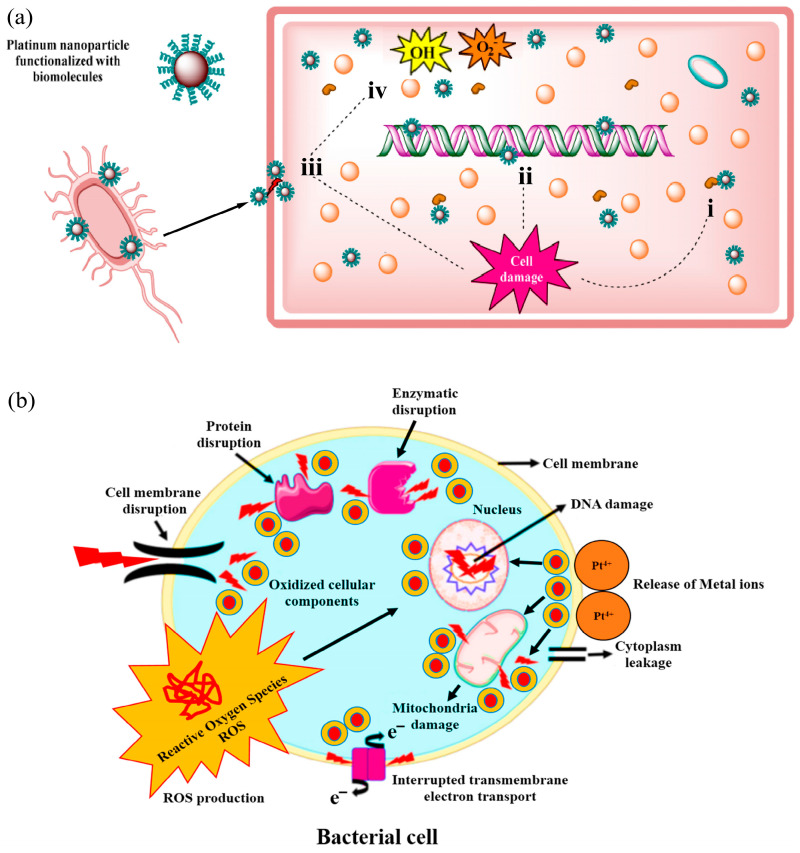
(**a**) Typical mechanisms of action of PtNPs against bacterial cells: i. denaturation of essential enzymes, ii. DNA damage, iii. cell lysis, and iv. production of hydroxyl radicals (OH) and superoxide (O_2_^−^). Reproduced under the terms of the Creative Commons CC-BY license [12]. Copyright 2022, the authors, published by MDPI. (**b**) Schematic illustration of the antibacterial activity of Pt-based active ingredients. Reproduced with permission [115]. Copyright 2022, Elsevier.

In addition to their anti-inflammatory effects, Pt-based materials also exhibit potent antimicrobial effects that are crucial for inhibiting the growth of skin pathogenic bacteria, thus improving the overall health of the skin. The antimicrobial action of PtNPs involves enzyme denaturation, DNA damage, cell lysis, and the production of ROS, such as hydroxyl and superoxide radicals (Figure 6a,b) [137]. These ROS can induce oxidative stress within microbial cells, leading to lipid peroxidation, protein alterations, enzyme inhibition, and RNA/DNA damage, which can cause cell death or significant mutations at sub-lethal doses [138,139]. Studies have shown that PtNPs can disrupt the integrity of bacterial cell membranes, leading to visible morphological changes such as depressions, perforations, and ruptures in bacterial cell walls [140]. These structural damages are typically accompanied by visible signs of cellular debris and leaked intracellular materials under microscopic observations.

**Figure 7:**
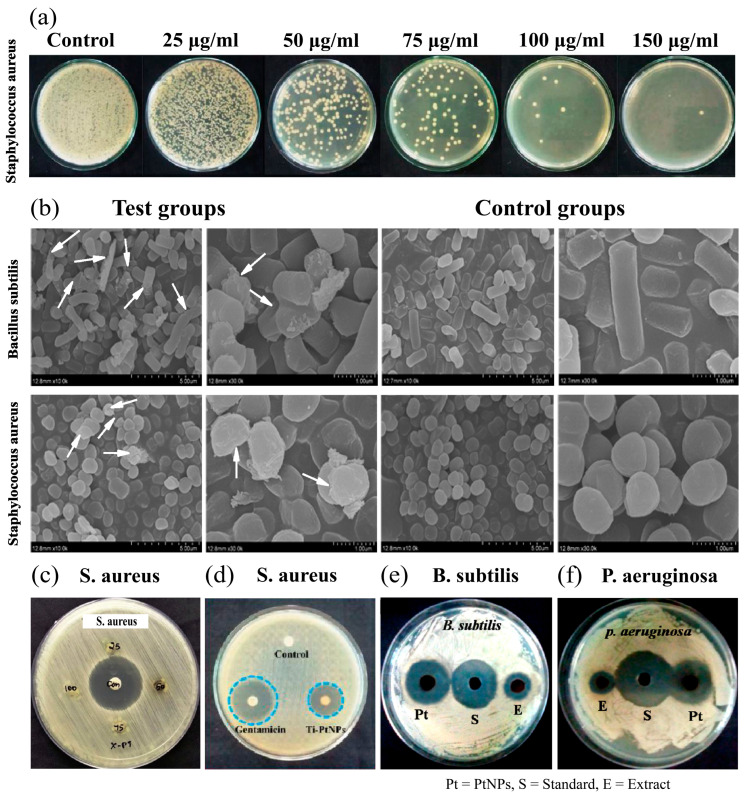
Antibacterial properties of Pt-based materials. (**a**) The antibacterial activity of PtNPs (biosynthesized using *Tragia involucrata* leaf extract) mediated using agar bioassay against *Staphylococcus aureus*. Reproduced with permission [111]. Copyright 2020, Elsevier. (**b**) Morphological changes in *Bacillus subtilis* and *Staphylococcus aureus* before and after treatment with PtNPs. White arrows indicate areas on bacterial surfaces showing depression, perforation, and rupture due to exposure to PtNPs. Reproduced under the terms of the Creative Commons CC-BY license [140]. Copyright 2022, the authors, published by MDPI. (**c**) Antibacterial study of PtNPs against *Staphylococcus aureus*. Concentration measured in μg/well with Azithromycin as the control at a concentration of 30 μg/well. Reproduced with permission [146]. Copyright 2019, Elsevier. (**d**) The antibacterial activity of PtNPs mediated by disk dilution methods against *Staphylococcus aureus*. Reproduced with permission [111]. Copyright 2020, Elsevier. (**e**,**f**) Antibacterial activity of green-synthesized PtNPs against two highly drug-resistant bacteria, *B. subtilis* and *P. aeruginosa*, compared to plant extract and a standard antibiotic. Pt = PtNPs, S = standard, and E = extract. Reproduced with permission [137]. Copyright 2016, Elsevier.

In recent years, with the increasing bacterial drug resistance, there has been an urgent need for alternative therapeutic agents that are non-toxic to humans but effective against pathogenic microorganisms. PtNPs, with their significant negative zeta potential, effectively disrupt bacterial cell integrity, offering a promising solution to enhance antimicrobial activity and combat microbial resistance [141]. Recent studies have highlighted the effectiveness of PtNPs against both Gram-positive and Gram-negative bacteria (Figure 7) [142]. These bacteria are responsible for severe infections affecting the skin, respiratory system, and other systemic areas, especially in immunocompromised patients [143]. Note that *Staphylococcus aureus*, *Staphylococcus epidermidis*, *Pseudomonas aeruginosa*, *Coagulase-negative staphylococci*, and various *Bacillus* species are predominant pathogens in skin and soft tissue infections, with Gram-positive *cocci* being prevalent, followed by Gram-negative *bacilli* [144]. Boomi et al. [145] reported that polyaniline/Ag-Pt nanocomposites (average size 2–5 nm) can inhibit the growth of *Staphylococcus aureus*, showing enhanced antimicrobial activity compared to pristine polyaniline, highlighting the crucial antimicrobial role of platinum. Tahir et al. [137] showed that biosynthesized PtNPs (2–7 nm in size) significantly inhibited the growth of both Gram-positive *Bacillus subtilis* and Gram-negative *Pseudomonas aeruginosa*, with zones of inhibition of 18 ± 0.8 mm and 15 ± 0.5 mm, respectively (Figure 7e,f). Note that the minimum inhibitory concentrations (MIC) of PtNPs were 53.2 μg/mL and 62.5 μg/mL, respectively. Kumar et al. [146] demonstrated that PtNPs at 100 μg/well effectively inhibited *Staphylococcus aureus* in well diffusion assays (Figure 7c). Selvi et al. [111] synthesized PtNPs using *Tragia involucrata leaf* extract, and the bimetallic nanoparticles significantly suppressed the growth of *Staphylococcus aureus*. The agar bioassay showed a concentration-dependent decrease in bacterial colonies, with the greatest inhibition at 150 μg/mL (Figure 7a), where the zone of inhibition against *Staphylococcus aureus* was found to be 19.6 ± 1.0 mm (Figure 7d). Liu et al. [140] synthesized PtNPs (average size 84.67 ± 5.28 nm) using *Cordyceps flower* extract. They observed significant morphological changes in *Bacillus subtilis* and *Staphylococcus aureus* that were exposed to PtNPs, including indentations, perforations, and ruptures, accompanied by visible cellular debris and intracellular material leakage around the bacteria (Figure 7b). These results confirmed that PtNPs could effectively inhibit bacteria associated with skin infections. However, while PtNPs can effectively inhibit bacterial growth, the zones of inhibition were generally smaller than those achieved with standard antibiotics such as Gentamicin, e.g., 28.8 ± 0.6 mm for Gentamicin versus 19.6 ± 1.0 mm for PtNPs against *Staphylococcus aureus*, as shown in Figure 7d. This suggests that enhancing the antimicrobial effects of Pt-based materials to match or surpass those of standard antibiotics remains a significant challenge for future research.

Aside from antimicrobial activity, the toxicity profile of PtNPs is also an important aspect that must be considered for their practical applications. Ahmed et al. [147] synthesized PtNPs with strong antimicrobial activity using pectin and sodium borohydride as capping and reducing agents. Their studies indicated that PtNPs could effectively reduce the viability of bacterial cells by inducing ROS production [147]. More importantly, they explored the optimal exposure dose of PtNPs by injecting 10 mL of PtNPs at various concentrations and showed that 0.1 mM was the optimal concentration based on the mortality rate at 48 h. PtNPs at such a concentration were found to be non-toxic to zebrafish during in vivo experiments, thus improving survival rates of infected zebrafish during antimicrobial treatment. In another study, Mukherjee et al. [148] demonstrated the biocompatibility of polyethylene glycolized colloidal platinum nanoparticles in hemolysis assays and confirmed their safety in mice at therapeutic doses of 10 mg/kg body weight.

Research has shown that the antimicrobial activity and toxicity of PtNPs, including their bimetallic variants such as AuPtNPs, are significantly influenced by their physicochemical properties, such as size, shape, surface modifications, and coatings [149]. These properties can affect various mechanisms for bacterial destruction, though the exact antimicrobial mechanisms remain unclear and controversial [96]. For example, Zhao et al. [150] found that while single-component metal nanoparticles lacked toxicity and antimicrobial properties, bimetallic AuPtNPs were effective against a broad range of bacteria. Their study revealed that the antimicrobial activity against *Escherichia coli* likely involved disrupting microbial cell membranes and increasing intracellular adenosine triphosphate (ATP) levels without generating ROS. These nanoparticles showed minimal toxicity in human umbilical vein endothelial cells, suggesting potential safety in humans, although the non-toxicity may not be universal across all cell types. In contrast, Nejdl et al. [151] found that PtNPs were toxic to both *Staphylococcus aureus* and human foreskin fibroblasts, with the toxicity mechanism linked to DNA damage. Similarly, Gholami et al. [152] demonstrated that fungi-biosynthesized PtNPs exhibited both antimicrobial properties and toxicity toward human hepatocellular carcinoma (HepG2) cells, whereas Brown et al. [153] showed no cytotoxicity to HepG2 cells at concentrations of PtNPs less than 20 μg/mL over a period of 1 h. These findings suggest that the antimicrobial efficacy and cytotoxicity of PtNPs are likely highly dependent on their specific design and synthesis.

Overall, while the antimicrobial mechanisms and toxic effects of PtNPs require further elucidation through toxicological studies, it is clear that platinum plays a critical role in human health, particularly in preventing skin diseases caused by bacteria and other microorganisms. Thus, it can be inferred that when PtNPs are used at appropriate sizes, concentrations, and exposure durations, they have the potential to effectively combat skin inflammation and bacterial growth without harming the host organism. This aspect makes PtNPs promising candidates for treating bacterial skin infections and associated inflammatory conditions.

### 3.3. Promotion of Collagen Synthesis

**Figure 8:**
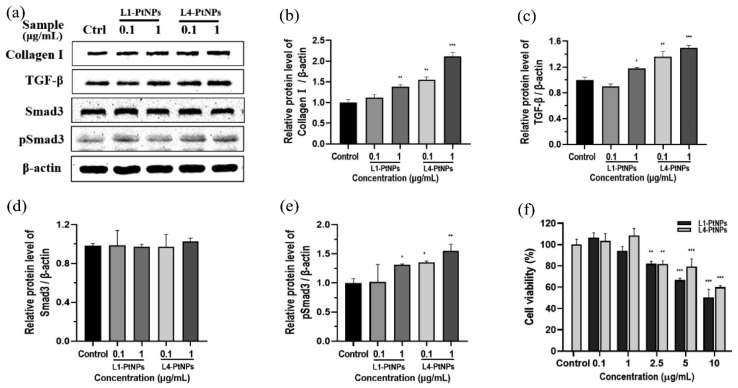
Platinum nanoparticles green-synthesized with a 1:1 ratio (L1-PtNPs) and a 1:4 ratio (L4-PtNPs) of H_2_PtCl_6_ to *N. tetragona* (L4-PtNPs) shown to promote collagen I by activating the TGF β/Smad signaling pathway. (**a**–**e**) Effects of L1-PtNPs and L4-PtNPs on collagen I, TGF β, Smad3, and pSmad3 expression in HFF-1 cells. (**f**) Effects of L1-PtNPs and L4-PtNPs on HFF-1 cell viability. Data are represented as the mean ± standard deviation from replicate experiments. * (*p* < 0.05), ** (*p* < 0.01), and *** (*p* < 0.001) represent different levels of statistical significance. Reproduced with permission [157]. Copyright 2022, Elsevier.

Collagen is an essential scaffold protein for maintaining skin elasticity and firmness, and a lack of sufficient collagen leads to signs of skin aging [154]. Pt-based materials, particularly PtNPs, have been identified as potential enhancers of collagen synthesis, which makes them active ingredients in anti-aging cosmetic formulations. Existing research suggests that the underlying mechanisms for promoting collagen synthesis by using Pt-based materials can be categorized into two primary pathways. The first mechanism involves the modulation of intracellular signaling pathways, such as the TGF-β/Smad signaling pathway. Transforming growth factor-beta (TGF-β) is a crucial activator of type I collagen synthesis in skin fibroblasts, with the Smad signaling pathway playing a dominant role in mediating the transcriptional response of collagen genes [155]. Studies indicate that PtNPs can stimulate this pathway, thereby enhancing collagen synthesis [156]. For example, Zhang et al. [157] demonstrated that PtNP treatment resulted in the upregulation of TGF-β expression and phosphorylation of Smad3 in HFF-1 cells, which are indicative of activated TGF-β/Smad signaling and enhanced synthesis of type I collagen (Figure 8). It was also found that PtNPs derived from chloroplatinic acid (H_2_PtCl_6_) and *Nymphaea tetragona* (*N. tetragona*) flower extract can effectively promote type I collagen synthesis and exhibit relatively high TGF-β/Smad pathway activity depending on the ratio between H_2_PtCl_6_ and *N. tetragona*.

In addition, oxidative stress plays a significant role in collagen degradation. The antioxidant properties of PtNPs, as mentioned above, can mitigate oxidative stress and provide indirect protection against collagen breakdown. For example, a mixture of palladium and platinum nanoparticles can prevent skin atrophy by reducing oxidative damage, thereby further inhibiting the downregulation of type I collagen [134]. However, for PtNPs to promote collagen synthesis effectively, they must penetrate the stratum corneum and reach the viable dermis. Thus, transdermal delivery methods become crucial. In cosmetic applications, PtNPs need not only to traverse the skin barrier effectively to deliver their benefits but also to be safely eliminated from the body without adverse effects. Here, the stratum corneum serves as the primary barrier to nanoparticle penetration. The routes for substance transport through the stratum corneum include the transcellular pathway, intercellular pathway, and transappendageal pathway [158].

Mauro et al. [159] investigated the dermal absorption of PtNPs and rhodium nanoparticles (RhNPs) using human skin layers, i.e., epidermis and dermis, with both intact and damaged skin barriers. They found that while platinum in nanoparticle form could penetrate both intact and damaged skin, no traces of the metal were detected in the receiving solution, suggesting that strong interactions between the nanoparticles and skin constituents (cells and extracellular matrix) may prevent platinum migration. By contrast, RhNPs penetrated damaged skin in small amounts after 24 h but failed to penetrate intact skin. Moreover, the metal content was higher in damaged skin compared to intact skin, twice as much for platinum and 17 times as much for rhodium, indicating that PtNPs have better penetration abilities compared to RhNPs and that skin lesions enhance platinum retention in the skin. That is to say, the condition of the skin (intact or damaged) is a crucial factor in the penetration efficacy of PtNPs, with damaged skin providing a weakened barrier more susceptible to nanoparticle penetration.

In addition to the state of the skin, factors such as particle size, surface charge, surfactant coatings, and the use of carriers and penetration enhancers can also influence the degree of skin penetration of PtNPs [158]. Generally, smaller nanoparticles are more likely to penetrate the skin [160], while the surface chemistry of nanoparticles, including hydrophilic and hydrophobic coatings and surface charge, can also affect their interactions with the skin. Surfactants on nanoparticle surfaces can improve dispersion and reduce friction, facilitating penetration. The lipophilic–hydrophilic gradient between the stratum corneum and the living epidermis dictates that nanoparticles must achieve an appropriate hydrophilic–lipophilic balance (HLB) to effectively penetrate the skin [160]. Given the negatively charged surface of the skin under physiological conditions, positively charged nanoparticles may adhere more strongly, leading to localized high concentrations, whereas anionic nanoparticles might be repelled [161]. Additionally, certain chemicals used as carriers or penetration promoters can enhance skin penetration. For example, the hydrophobicity of transdermal carriers has been shown to improve skin penetration [162,163].

**Figure 9:**
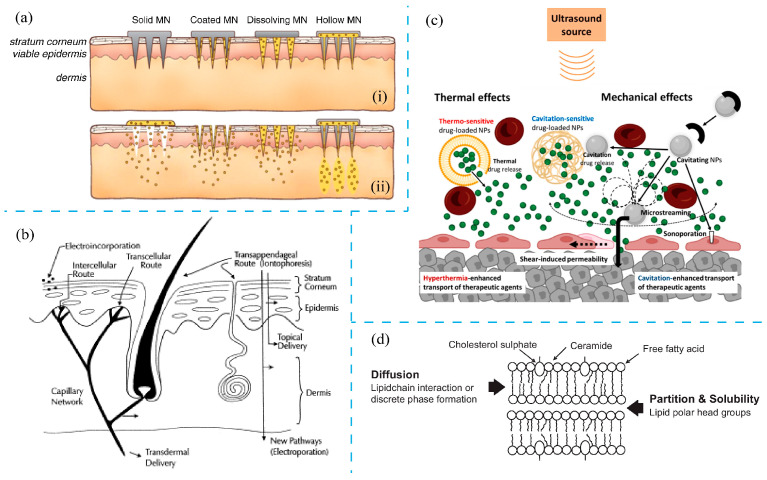
(**a**) Methods of drug delivery to the skin using microneedles (MNs). Microneedles are first applied to the skin (**i**) and then used for drug delivery (**ii**). Solid microneedles are used as a pretreatment, after which the drug can diffuse through residual holes in skin from a topical formulation (solid MNs). After insertion of drug-coated microneedles into the skin, the drug coating dissolves off the microneedles in the aqueous environment of the skin (coated MNs). Drug-loaded microneedles are made of water-soluble or biodegradable materials encapsulating the drug that is released in the skin upon microneedle dissolution (dissolving MN). Hollow microneedles are used to inject liquid formulations into the skin (hollow MNs). Reproduced with permission [173]. Copyright 2012, Elsevier. (**b**) A schematic showing the pathways of topical and transdermal delivery, including electrically assisted delivery by iontophoresis, electroporation, or electroincorporation. Reproduced with permission [168]. Copyright 1999, Elsevier. (**c**) Schematic of various mechanisms of ultrasound-triggered release of drug agents from nanoparticles as well as drug transport. Reproduced with permission [174]. Copyright 2023, Elsevier. (**d**) Schematic of diffusion–partition–solubility actions of skin penetration enhancers. Reproduced with permission [175]. Copyright 2013, Elsevier.

Considering the critical factors affecting the transdermal capabilities of PtNPs, dermatologists often supplement natural penetration abilities with additional delivery mechanisms (Figure 9). For example, by utilizing microneedling (Figure 9a) or radiofrequency techniques, the barrier efficacy of the stratum corneum can be bypassed through the creation of microchannels in the skin, thereby enhancing nanoparticle delivery. Studies demonstrate that microneedles significantly facilitate the penetration of nanoparticles [164,165]. Zhang et al. [166] found that microneedles could improve the penetration and distribution of poly(d,l-lactic-co-glycolic acid) (PLGA) nanoparticles for intradermal delivery. Similarly, Birchall et al. [167] observed that radiofrequency techniques generate microchannels that allow for the epidermal delivery of nanoparticles up to 100 nm in diameter. After applying a radiofrequency microchannel generator (ViaDerm™), they successfully created channels in human breast skin, enhancing its permeability. In addition, mechanical forces (e.g., bending and massaging), transdermal potentials (e.g., electrophoresis, iontophoresis, and electroporation [168,169]) (Figure 9b), and diffuse mechanical stresses, such as ultrasound [170,171] (Figure 9c), can also help to increase skin permeability. These techniques provide external impetus for nanoparticles to penetrate the stratum corneum barrier more effectively. Moreover, chemical enhancers that alter the stratum corneum lipid structure can further facilitate skin penetration (Figure 9d) [172].

Optimizing transdermal delivery methods requires considering how Pt-based materials interact with target cells and overcome the skin barrier to reach these cellular targets. Despite the limited reports on the skin permeability of PtNPs, analogies are often drawn with gold nanoparticles, another group of precious metals known for their skin penetration capabilities. According to Chen et al. [176], gold nanoparticles can penetrate into the deeper layers of the skin and deliver the drug. Their permeability is influenced by factors such as particle size, surface properties (including hydrophilicity and charge), and shape, which collectively determine their permeation behavior through intercellular and transcellular routes and across skin accessory structures [177]. Similarly, with appropriately designed platinum nanoparticles, it is possible to achieve dermal permeability. On the other hand, the matrix of hydrophilic, lipophilic, and emulsifying agents in skincare products can modify skin conditions to enhance the absorption of platinum nanoparticles. For example, hydrophilic permeation enhancers can increase the hydration of the stratum corneum to facilitate the penetration of hydrophilic ingredients, while lipophilic enhancers such as ethanol can disrupt the lipid structure of the stratum corneum, thus improving its permeability [178]. Such strategic product design allows platinum nanoparticles to more easily bypass the stratum corneum and reach the active layers of the skin.

Overall, the ability of PtNPs to enhance collagen production holds significant promise for dermatology and skincare applications. By enhancing the transdermal ability of PtNPs, collagen levels can be increased, which further improves skin texture and reduces wrinkles, making them ideal ingredients for anti-aging skincare formulations. In addition, the dual functionality of PtNPs in stimulating collagen synthesis and protecting it through antioxidant activity provides a compelling case for their inclusion in dermatological products. These insights highlight the potential of PtNPs for developing new skincare products designed to enhance skin texture and reduce the appearance of wrinkles. However, further studies are still required to fully elucidate the underlying mechanisms through which PtNPs exert these effects, to improve transdermal efficiency, and to evaluate their safety and efficacy in topical applications.

### 3.4. Effects on Melanogenesis

**Figure 10:**
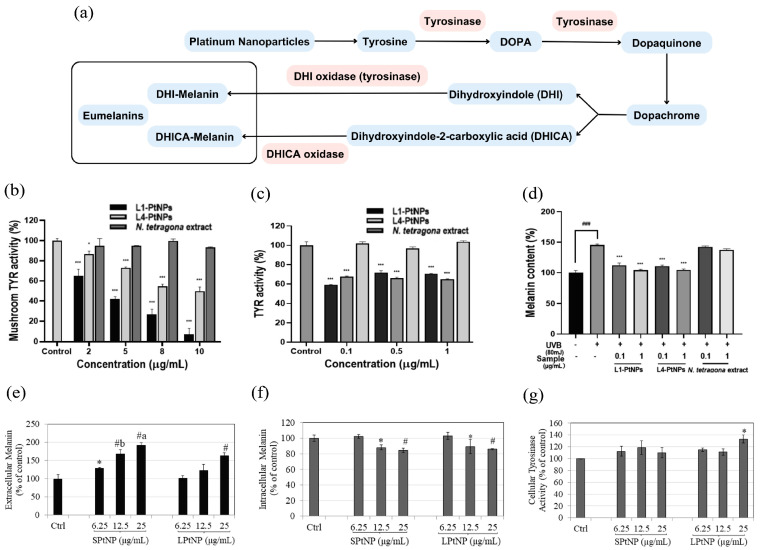
(**a**) Mechanisms by which PtNPs regulate melanogenesis by affecting tyrosinase activity. (**b**) Effects of PtNPs and *N. tetragona* extract on mushroom tyrosinase activity. (**c**) Effects of PtNPs and *N. tetragona* extract on tyrosinase activity in A375 cells. (**d**) Effect of PtNPs and *N. tetragona* extract on ultraviolet B (UVB)-induced melanin biosynthesis in A375 cells. Data are represented as the mean ± standard deviation from replicate experiments. Statistical significance is denoted by: # *p* < 0.05, ### *p* < 0.001 compared to the control; * *p* < 0.05, *** *p* < 0.001 compared to UVB-treated cells. Reproduced with permission [157]. Copyright 2022, Elsevier. (**e**) Extracellular and (**f**) intracellular melanin levels in cultures of MNT-1 cells treated for 72 h with different concentrations of smaller (SPtNP) and larger PtNPs (LPtNP). Statistical analyses were performed using one-way analysis of variance (ANOVA) followed by Tukey’s test. # *p* < 0.01 and * *p* < 0.05 versus control; #b indicates *p* < 0.001 compared to LPtNP at 12.5 µg/mL, and #a indicates *p* < 0.05 compared to LPtNP at 25 µg/mL. (**g**) Cellular tyrosinase activity in MNT-1 human melanoma cells treated for 72 h with different concentrations of PtNPs (* *p* < 0.05, assessed by one-way ANOVA followed by Tukey’s test). Data are represented as the mean ± standard deviation from at least two independent experiments. Reproduced under the terms of the Creative Commons CC-BY license [181]. Copyright 2020, the authors, published by MDPI.

The modulation of melanogenesis is an important property of Pt-based materials that has received great interest in dermatology. Melanin, the pigment produced by melanocytes within the skin, mainly functions to absorb ultraviolet light, thereby protecting the skin from harmful ultraviolet (UV) damage [179]. However, overproduction of melanin can lead to skin discoloration, freckles, and other pigmentation issues, making melanin regulation an important aspect of skincare and cosmetic applications [180]. It has been found that specific sizes and shapes of PtNPs can affect melanogenesis, offering new approaches for regulating skin pigmentation [181]. This capability is particularly valuable for applications such as skin whitening treatments and managing hyperpigmentation disorders. Research has suggested that PtNPs may regulate melanogenesis by affecting the activity of tyrosinase, a key regulatory enzyme in this process (Figure 10a) [182]. Note that tyrosinase, identified as the rate-limiting enzyme in melanogenesis, can catalyze the hydroxylation of L-tyrosine to L-dopa and the oxidation of L-dopa to dopaquinone, thereby determining the course of melanin synthesis [183]. The ability to affect tyrosinase activity suggests that PtNPs can be utilized to alter skin pigmentation effectively.

Zhang et al. [157] found that the PtNPs synthesized using *N. tetragona* flower extract not only reduced mushroom tyrosinase activity in vitro but also mitigated tyrosinase activity and UV-induced melanogenesis in A375 human melanocyte cells, contributing to a whitening effect. As shown in Figure 10b, *Lobelia* extract had no significant inhibitory effect on tyrosinase activity, while PtNPs showed a strong inhibitory effect. On the other hand, while *N. tetragona* extract alone did not significantly inhibit intracellular tyrosinase activity in A375 cells, platinum nanoparticles synthesized with a 1:1 ratio (L1-PtNPs) and a 1:4 ratio (L4-PtNPs) of H_2_PtCl_6_ to *N. tetragona* exhibited significant inhibitory effects at concentrations of 0.1, 0.5, and 1 μg/mL, with no significant differences between the two ratios (Figure 10c). In addition, it was found that *L. longifolia* extract did not reduce melanin synthesis, whereas PtNPs significantly inhibited intracellular melanogenesis compared to UVB-treated control. This was consistent with their effects on tyrosinase activity, demonstrating their whitening effect. In addition, Goenka and Toussaint [181] observed a size-dependent effect of citrate-coated PtNPs on melanin formation, with smaller PtNPs (5 nm) inducing more extracellular melanin production (Figure 10e) and decreasing intracellular melanin (Figure 10f) compared to larger PtNPs (50 nm). It was found that larger PtNPs also enhanced intracellular tyrosinase activity, significantly increasing activity at 25 µg/mL compared to controls (Figure 10g), whereas smaller PtNPs did not affect tyrosinase activity at any tested concentration. While the mechanism driving the increase in melanin production by large particle size remains unclear, it is interesting that the effects of PtNPs on melanin secretion and synthesis were reversible when the nanoparticles were removed from the culture medium, suggesting that PtNPs could act as modulators of melanogenesis. These results highlight the promising applications of PtNPs in treating uneven skin pigmentation, enhancing sunless tanning, and potentially restoring gray hair.

**Figure 11:**
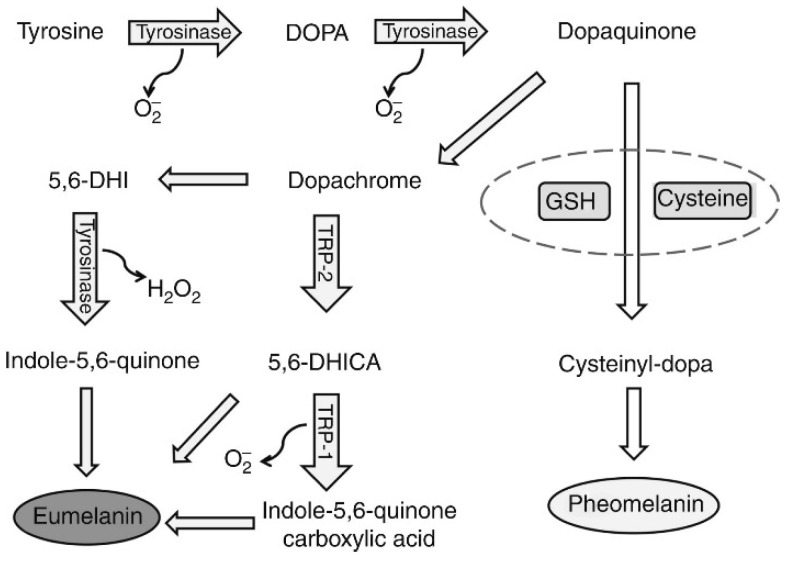
Illustration of the generation of ROS at various steps in the melanin synthetic pathway. Reproduced with permission [184]. Copyright 2014, Elsevier. Note: 5,6-DHI represents 5,6-Dihydroxyindole, 5,6-DHICA represents 5,6-dihydroxyindole-2-carboxylic acid, and GSH represents glutathione.

Studies have shown that oxidative stress is a key contributor that leads to increased melanin production [184]. Oxidative stress can disrupt melanocyte homeostasis and heighten susceptibility to ROS, particularly in epidermal melanocytes, which are stimulated by sunlight exposure, tanning, and post-inflammatory hyperpigmentation processes (Figure 11) [185,186,187,188]. The excellent antioxidant properties of platinum can reduce the extent of oxidative stress, thereby affecting melanin production. Yoshihisa et al. [21] found that PtNPs can reduce UV-induced skin inflammation and promote skin whitening by scavenging ROS and reflecting UV light. In vivo tests further revealed that gels containing platinum can effectively alleviate inflammation and phototoxic reactions from UV exposure, highlighting the potential of PtNPs as a photoprotective agent.

Given the ability of platinum to influence melanogenesis, there is substantial interest in its application in cosmetology for developing effective solutions for skin whitening and managing pathological pigmentary disorders. Continuous research aims to understand the specific pathways through which platinum affects melanin production and to evaluate the safety and efficacy of Pt-based active ingredients in dermatology and skincare applications.

## 4. Applications in Dermatology and Skincare

### 4.1. Platinum in Cosmetic Skincare

**Figure 12:**
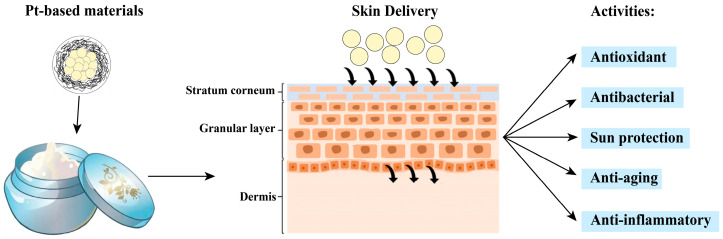
Schematic illustration of the topical application of Pt-based active ingredients (modified from ref. [81]). Reproduced with permission [95]. Copyright 2021, Elsevier.

Skin aging is characterized by dryness, loss of elasticity, texture changes, thinning, impaired barrier function, and the appearance of spots and wrinkles, driven by factors such as chemical exposure, pollution, stress, UV radiation, and natural wear and tear [189]. There are many personal care products in the market that claim benefits including anti-wrinkle and firming effects, moisturization, lifting, skin conditioning, and whitening. As aforementioned, Pt-based active ingredients, with controllable structure and physicochemical properties of the materials, offer versatile biological effects that can be useful in dermatology and cosmetics (Figure 12). These materials can promote skin cell metabolism, enhance skin elasticity and radiance, slow skin aging, smooth fine lines and wrinkles, and increase firmness. Additionally, the strong antioxidant properties of platinum can protect the skin from environmental pollutants and UV radiation damage, offering a multifunctional approach to combat skin aging.

Platinum is currently employed as an active ingredient in various skincare products, including masks, serums, and creams (Table 3). High-end brands, including La Prairie, Cada Suissesse, and Skin Advanced, feature Pt-based active ingredients in their skincare lines, utilizing their potent anti-aging properties to enhance skin elasticity and firmness and reduce visible signs of aging such as wrinkles and fine lines (Table 4). Each brand uses unique technologies or ingredient combinations to enhance product efficacy and target specific skin issues (Table 4). For example, La Prairie employs platinum peptides and a unique revitalizing complex in its formulations to diminish fine lines and improve skin elasticity. Skin Advanced utilizes a non-nano-sized platinum liposome technology to boost antioxidant levels, increase skin hydration, promote barrier function, and alleviate redness. This approach also aims to minimize potential safety concerns associated with the skin penetration of nanoparticles. Products containing platinum are deemed clinically safe and are permitted for market release only after meeting stringent local regulations, including a toxicological risk assessment that requires detailed toxicological data on all ingredients. As deeper insights into Pt-based materials are gained continuously, we believe that the corresponding regulations are likely to be updated to reflect the latest scientific discoveries. It is worth mentioning that these products typically occupy a luxury market position, emphasizing unique formulations and advanced technologies to attract consumers and maintain a competitive edge.

Furthermore, the integration of Pt-based active ingredients in skincare products may offer additional relaxation benefits beyond typical dermatological improvements. A study by Kokubo et al. [190] measured electroencephalograms of 16 healthy adult women who regularly use cosmetics, finding that products containing platinum provided noticeable relaxation effects. It has been suggested that platinum could remain on the skin surface rather than being absorbed, thereby influencing the electronic state of skin with its −40 mV potential [190]. However, this speculation requires further validation, as the observed relaxation effects could be influenced by a variety of factors, including the sensory experience of applying the product itself. Despite this, such preliminary evidence suggests that platinum can potentially offer psychological relaxation in addition to its physical skincare benefits, presenting a unique direction for future research and product development.

### 4.2. Platinum in the Treatment of Skin Inflammation

**Figure 13:**
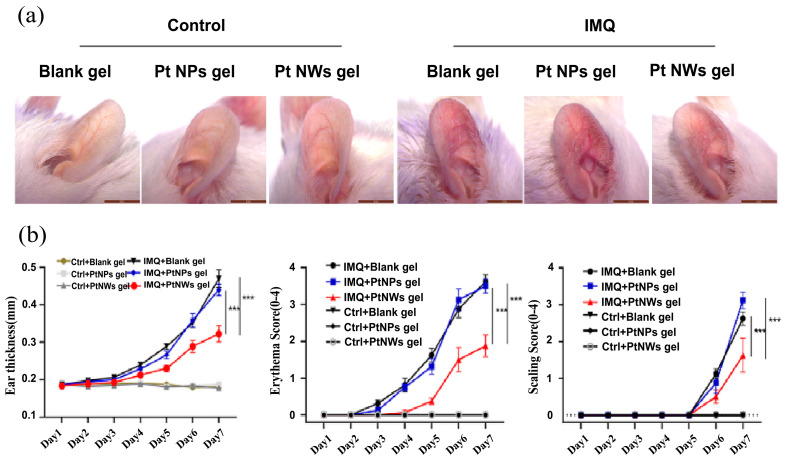
Treatment of psoriasis-like skin inflammation in mice using platinum nanowire (PtNW) gel. (**a**) Representative phenotypic presentation of mouse ears treated with blank gel, PtNP gel, or PtNW gel. (**b**) Clinical scores for ear thickness, scaling, and erythema in mice with psoriasis-like symptoms. Error bars represent the standard error of the mean. *** (*p* < 0.001) represent different levels of statistical significance, assessed using a two-way ANOVA test. Reproduced with permission [198]. Copyright 2024, Elsevier.

Inflammatory skin diseases, such as psoriasis and rosacea, are characterized by chronic, immune-mediated inflammation [191,192]. While the exact causes of these conditions remain unclear, oxidative stress and inflammation are known to play significant roles [193,194]. Increased production of ROS is a key mechanism driving these dermatological issues [193,195]. There is growing evidence that suggests that patients with these conditions often exhibit elevated oxidative stress markers and reduced antioxidant levels [129,196,197]. Therefore, therapies that can enhance or mimic antioxidant enzymes could be helpful in treating inflammatory skin diseases. As aforementioned, platinum can be used as novel nanoenzymes [198] to mitigate ROS with its excellent antioxidant properties, thus alleviating inflammatory skin diseases. Studies have demonstrated that platinum nanowires (PtNWs) can reduce inflammatory responses in mouse models of psoriasis-like skin inflammation induced by imiquimod (IMQ) by inhibiting oxidative stress and activating inflammatory cytokines [198]. It was found that mice treated with topical platinum nanowire gel displayed reduced ear thickening, scaling, and erythema compared to controls treated with IMQ alone (Figure 13a,b). Additionally, topical application of platinum nanowires or PtNP gel did not induce adverse changes in models without IMQ induction (Figure 13b).

On the other hand, acne, a common inflammatory skin condition, is typically caused by excessive sebum secretion and follicular blockage [199]. Acne lesions, ranging from comedones to nodules and cysts, often lead to significant dermatological distress [200]. Traditional treatments for acne, including systemic antibiotics and topical retinoids, are effective but limited by issues such as drug resistance and risk of teratogenicity [201]. This has led to the exploration of novel treatments such as platinum-based photothermal therapy, which was originally developed for cancer treatment. In photothermal therapy, nanoparticles produce heat in response to specific light wavelengths, triggering cell necrosis and apoptosis through cytotoxic photothermal heating and ROS generation [202,203]. PtNPs, known for their surface plasmon resonance effect, are being studied as photothermal agents and photosensitizer carriers in photothermal therapy [204]. When used with ultrasound devices and laser pulses, these nanoparticles can selectively target and thermally damage sebaceous glands deep within the skin, minimizing surface damage. A study by Chou et al. [205] demonstrated the effectiveness and safety of this approach in a case study with five acne patients. After treatment, a significant reduction in acne lesions was observed, and red fluorescence was reduced in all five patients compared with baseline. It was found that PtNPs, which were selectively absorbed through the skin pores around sebaceous glands and hair follicles, can significantly improve acne lesions without severe side effects or recurrence. The effectiveness of PtNPs in photothermal therapy is attributed to their high photothermal conversion efficiency, their ability to be functionalized for drug delivery, and their potent antioxidant effects, which help minimize collateral damage during treatment [134]. Moreover, these nanoparticles also exhibit high energy absorption at near-infrared wavelengths, enabling them to release heat that can effectively destroy problematic cells nearby [28]. Despite these advantages, platinum-based photothermal therapy is more costly compared to conventional acne treatments (e.g., systemic antibiotics or topical vitamin A analogs) due to the need for specialized equipment and expensive materials. Further studies are needed to fully establish the efficacy and broader applications of this promising technology.

Studies have shown that combining platinum-based photothermal therapy with moisturizing and soothing ingredients can significantly enhance skin health, as evidenced by positive results in facial stinging and discomfort testing [206,207]. In Japan, a combination of gentle cleansing milk with a fortified moisturizing cream (referred to as “combination skincare”) has proven effective in treating mild acne and sensitive skin, reducing facial stinging and discomfort [208]. This suggests that platinum could be effectively integrated into such skincare combinations, potentially developing non-irritating cosmetics that offer moisturizing benefits. Furthermore, platinum may help alleviate acne symptoms by reducing inflammation and inhibiting bacterial growth, although its irritability and safety profiles still require a thorough evaluation. Overall, while current studies on Pt-based materials for treating skin inflammation are still preliminary and lack extensive clinical trials, they suggest promising directions for future research into the benefits of platinum in dermatological treatments.

## 5. Safety and Sustainability of Pt-Based Materials

While Pt-based materials have shown great potential for various dermatological applications, their safety and sustainability are still an issue of great concern. Consequently, there is a need for comprehensive studies to evaluate the biological behavior and potential toxicity of these materials or nanomaterials upon skin contact, ensuring their safe use in dermatology.

In existing studies, platinum is predominantly utilized in the form of nanoparticles, which exhibit excellent physicochemical properties. However, as aforementioned, traditional chemical and physical synthesis methods for Pt-based materials often involve energy-intensive processes and the use of hazardous chemicals, which not only raise concerns about environmental impact but also pose potential safety risks. Residual unreacted chemicals and hazardous by-products may adhere to the surfaces of nanoparticles, making them less suitable for applications related to skin health and cosmetics [209,210,211]. To overcome these drawbacks, researchers have turned to eco-friendly biosynthesis methods for producing Pt-based materials, including PtNPs. This approach utilizes a variety of bio-sourced entities, including bacteria, fungi, enzymes, plants, and algae [212,213,214,215,216], and extends to other biological resources, such as egg yolk, goat milk, honey, and bovine serum albumin [4]. The biosynthesis of PtNPs not only has short reaction times and low energy consumption but also provides safer manufacturing conditions [64]. These advantages make biosynthesized PtNPs particularly suitable for pharmaceutical and biomedical applications due to their biocompatibility, environmental sustainability, and cost-effectiveness [209,217]. Currently, vegetable derivatives are described in many studies for synthesizing PtNPs; e.g., Raut et al. [218] reported a rapid preparation of monodispersed spherical 1–6 nm PtNPs in room temperature aqueous solution using totalized asparagus root extracts, which is a clean, fast, and environmentally friendly synthesis method.

However, although many studies have demonstrated that plant extracts are effective bio-reducers and stabilizers as a “green reagent” and the use of such bio-assisted procedures is promising for health applications, so far, methods are still inefficient in terms of morphology control. Since the reactions are completely dependent on the specific phytochemicals involved, they usually do not allow fine control of the nanoparticle properties. Therefore, in order to obtain well-defined nanoparticles of different shapes, attention has been directed to some common reducing agents for nanoparticle synthesis, such as ascorbic acid and citrate, which are also considered “green reagents” and which, by tightly controlling the purity of solvents and reagents, allow the synthesis of biocompatible PtNPs as well [219,220,221,222]. In the preparation of PtNPs using citrate, the citrate ion is responsible for both the reduction of the metal salt and the stability of the nanoparticles formed in aqueous solution. Among them, trisodium citrate is the sodium salt of citric acid, a natural compound found mainly in citrus fruits [223]. Therefore, trisodium citrate can be considered ecologically safe and biodegradable. Lin et al. [224] prepared PtNPs with an average size of 2–3 nm and a distribution of about ±2 nm by methanol reduction using sodium citrate as a stabilizer. It was found that the particle size and sol–gel stability could be controlled by changing the ratio of Pt/citrate. In the absence of citrate, the chemical conversion of Pt (IV) to Pt (0) was faster, and the particles grew by aggregation to form larger agglomerates and precipitates. In the presence of citrate, the interaction of citrate with the newly formed platinum particles reduced the growth tendency of the platinum particles and led to a decrease in the number of Pt-Pt coordination sites and a slowing down of the sol–gel formation process. In addition, ascorbic acid, also known as L-hexuronic acid, which is a form of vitamin C, is a naturally occurring organic compound with antioxidant properties and can be considered a green reagent due to its environmentally friendly and non-toxic properties [225]. A size-tunable octahedral platinum nanocrystal was synthesized by Moglianetti et al. [226]. The synthesis required sodium citrate and ascorbic acid with a finely controlled rate of reduction in an aqueous environment. L-ascorbic acid and sodium citrate play an important role in the reduction of precursors and stabilization of nanoparticles, and from a catalytic point of view, they both only bind to the surface of the platinum nanocrystals through their carboxyl and hydroxyl groups. This binding is weak, and thus, they can be easily removed by sodium hydroxide while stabilizing the particles in solution and preventing aggregation. In conclusion, this synthetic approach using ascorbic acid and citrate as reducing or stabilizing agents ensures the precise control of size, shape, and catalytic properties, as well as reasonable yields, and this synthetic strategy allows for easier functionalization of the nanoparticle surfaces, which is essential for the design of PtNPs for dermatological applications.

While biosynthesis is considered a greener and potentially safer method for producing Pt-based materials, it does not guarantee that these materials, especially nanomaterials, are non-toxic and safe for biological use. The use of PtNPs in bio-related applications remains a subject of large debate due to unresolved questions regarding their toxicological properties. For example, Claudia et al. [227] found that 70 nm citrate-encapsulated PtNPs led to approximately 25% cytotoxicity in the HepG2 liver cell model at a high concentration of 100 μg/mL, whereas at a lower concentration of 25 μg/mL, the nanoparticles induced cellular stress. This finding suggests that while acute exposures to PtNPs are not significantly toxic, prolonged cellular interactions could lead to long-term health consequences. In addition, Gehrke et al. [228] observed that different sizes of PtNPs (<20 nm, <100 nm, and >100 nm) deposited on a β-cyclodextrin matrix showed no cytotoxicity to HT29 cells up to a concentration of 1000 ng/cm^2^ (by SRB/LDH/WST-1 assay). However, they found that PtNPs tended to bind with DNA in a concentration- and time-dependent manner. Although neither cytotoxicity nor ROS activation to HT29 was detected with high platination of DNA, the potential for damage remains unclear and should not be disregarded, especially considering oral uptake. Moreover, Rehman et al. [229] reported that 2.4 nm citrate-encapsulated PtNPs were not cytotoxic to RAW 264.7 cells at concentrations below 1000 μM platinum, highlighting the complex interactions of PtNPs with biological systems. The above findings show that acute cytotoxicity results can vary significantly due to many factors, such as cell type, the size of PtNPs, concentration, surface coating, etc. In addition, regarding the long-term toxicological profiles of PtNPs, there are two potential mechanisms that need future attention. Firstly, PtNPs may induce ROS production and inflammation under specific conditions. Secondly, Pt ions may dissociate from nanoparticles through complex biological reactions and interact with cellular components. These potential mechanisms could lead to non-negligible health risks over time.

Besides the potential chemical toxicity of Pt-based materials, attention should also be paid to the accumulation of nanoparticles in vivo due to their high surface-area-to-volume ratio. Thus, understanding the biodistribution and pharmacokinetics of PtNPs is essential. Mukherjee et al. [148] synthesized spherical PtNPs coated with PEG_6000_ with a core size of 2–10 nm and a hydrodynamic diameter of 40–45 nm. Injections given to mice at a low dose (10 mg kg^−1^ b.w.) and a high dose (50 mg kg^−1^ b.w.) were examined through blood hematology, serum biochemical analysis, and tissue histopathology. It was found that while the low dose of PtNPs was safe and biocompatible, the high dose showed slight hepatotoxicity and histopathological alterations in kidney and brain tissues. Biodistribution studies indicated that PtNPs accumulated in major organs, including the spleen, liver, heart, and kidneys, with them being present predominantly in the spleen, which may result in long-term toxicity. The total amount of accumulated platinum in these organs was found to be approximately 10%, with the remainder excreted through feces and urine depending on the nanoparticle size. In another study, Brown et al. [153] synthesized hydrophobic, spherical PtNPs (core size 2–4 nm) and created PEGylated PtNPs with a hydrodynamic diameter of 50–150 nm through the covalent conjugation of phosphoethanolamine lipid and polyethylene glycol (DSPE-PEG). Various doses of PtNPs (5/10/15/20 mg/kg) given to mice led to no toxicity or abnormal levels. Biodistribution studies revealed significant accumulation in the liver (20.26%) and spleen (6.56%) after 24 h exposure, with no platinum detectable in plasma, indicating rapid excretion from the system.

It is important to note that small amounts of PtNPs can be retained in the body and may take a long time to be fully excreted once they enter systemic circulation, with their long-term toxicity still not fully understood. The biodistribution and pharmacokinetics of PtNPs are rather complex and can be significantly influenced by their physicochemical properties. It is advisable that Pt-based materials or active ingredients designed for cosmetic products be carefully engineered or formulated to avoid entering systemic circulation.

**Figure 14:**
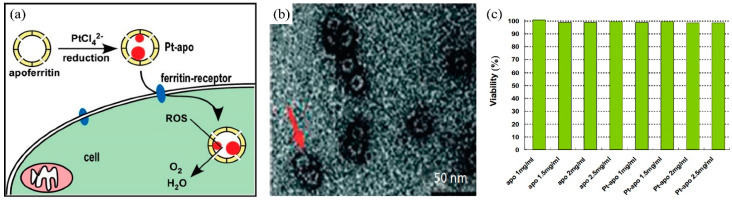
(**a**) PtNPs were stabilized within apolipoprotein cages to enable cellular uptake of PtNPs without disrupting biological systems. (**b**) TEM images of apoferritin-encapsulated PtNPs negatively stained with 1.5% uranyl acetate. The red arrow indicates an apoferritin cage that is either empty or contains very small PtNPs. (**c**) Cytotoxicity assay for apoferritin (apo) and Pt-apoferritin (Pt-apo) on Caco-2 cells, which were incubated with either apo or Pt-apo in culture medium for 1 h at 37 °C. Reproduced with permission [230]. Copyright 2010, American Chemical Society.

To mitigate potential toxicity, Zhang et al. [230] synthesized PtNPs within an apo-ferritin cage, which can enhance cellular uptake without harming biological systems such as lipid membranes or cellular proteins. These apo-encapsulated PtNPs showed effective ROS scavenging capabilities (Figure 14a,b) while, at the same time, being considered relatively safe due to their encapsulation. With a diameter of approximately 2 nm, these PtNPs encased in apoferritin protein (Pt-apo) increased cell viability in Caco-2 cells under oxidative stress at a high concentration of 1.0 mg/mL, suggesting an antioxidant protective effect. The Cell Counting Kit-8 assay showed no concentration-dependent toxic effects over the tested range, with cell viability reductions of less than 2% compared to untreated cells (assumed viability of 100%) and a standard deviation below 0.8% (Figure 14c). Nevertheless, the overall safety of platinum materials may depend significantly on their synthesis and surface properties. Ongoing research and clinical trials are essential to confirm the safety of Pt-based materials for dermatological use. The development of green biosynthesis methods and the use of benign encapsulation techniques could further enhance the sustainability and safety of Pt-based materials in dermatology.

In addition to their long-term toxicity, the environmental impact of Pt-based materials is a critical aspect of toxicological studies. Colombo et al. [231] demonstrated in vitro that platinum group elements (PGEs)–chloride species can form from vehicle exhaust and road dust under acidic conditions and in the presence of chloride ions [232,233]. Platinum has been identified as the most bioavailable of the PGEs, which can pose health risks even in trace amounts due to its toxicity and allergenic potential. Additionally, platinum-based cytostatic drugs excreted post-therapy often contaminate wastewaters, contributing to environmental pollution [234]. Over time, the gradual accumulation of Pt ions can harm plants and animals, eventually contaminating food chains [235].

From an environmental safety perspective, the percutaneous absorption of nanoparticles is crucial for assessing human exposure risks. Nanoparticles can enter the body through inhalation, ingestion, or dermal contact, especially when skin is damaged. This exposure risk is heightened by the use of medications or cosmetic formulations that contain nanoparticles [236]. The size of nanoparticles plays a significant role in their nanotoxicity; smaller nanoparticles are more likely to cross skin barriers and cell membranes, potentially entering organs, tissues, and cells [237]. Manikandan et al. [238] found that 5–6 nm PtNPs are non-cytotoxic, whereas in a different study, Konieczny et al. [239] reported that both 5.8 nm and 57 nm PtNPs could disrupt cellular metabolism in primary keratinocytes without affecting cell viability or migration. Smaller nanoparticles, however, were more likely to destabilize DNA and activate caspases [239].

Nanoparticles are predominantly found in stratum corneum and hair follicles, where their accumulation and potentially toxic effects might be mitigated by the shedding of stratum corneum, the outward flow of sebum, and the lack of viable cells. However, given the variability in skin types, application methods, and nanoparticle compositions, a more rigorous multidisciplinary approach is required to fully understand the mechanisms of nanoparticle penetration and interaction with the skin [160,240,241,242]. Additionally, certain Pt-based materials may induce neurotoxicity, especially with common chemotherapeutic agents such as oxaliplatin and cisplatin. The adoption of green synthesis methods or the use of neuroprotective agents (e.g., vitamin E, glutathione, amifostine, xaliproden, and venlafaxine) may help reduce this toxicity [71,243]. Overall, understanding the toxicological profiles of Pt-based materials is vital for their safe and sustainable development, yet current research is insufficient to fully elucidate these mechanisms.

## 6. Conclusions and Outlook

In this review, we provide an overview of recent studies on the biological effects and therapeutic potential of Pt-based active ingredients in dermatological and skincare applications. By presenting their synthesis methods and key properties, such as antioxidant, anti-inflammatory, antimicrobial, and collagen synthesis capabilities, we highlight the potential of these materials to address a variety of skin conditions as well as enhance skin aesthetics. These research advancements not only deepen our understanding of Pt-based materials but also open new possibilities for their applications in dermatology and skincare solutions. Looking forward, there are several promising directions for further development in this field:

(i) Exploring diverse forms of Pt-based materials for enhanced antioxidant properties: Maximizing the antioxidant capabilities of Pt-based materials is essential for dermatological applications. The biological interactions and efficacy of these materials are significantly influenced by their physical characteristics, such as shape, size, and form. Currently, our understanding of how these structural properties relate to biological effects is limited, complicating the development of specifically tailored Pt-based active ingredients. Future research should aim to optimize the catalytic and radical scavenging activities of various platinum forms, which can potentially lead to more effective anti-aging and skin-protective solutions.

(ii) Improving antimicrobial efficacy and addressing resistance: To surpass the effectiveness of conventional antibiotics and prevent antimicrobial resistance, advancements in the design and application of Pt-based materials are essential. This involves not only optimizing the size and shape of Pt-based materials for enhanced penetration and action against microbial biofilms but also developing strategies to minimize the potential risk of resistance development. Investigating the mechanisms of Pt-based materials’ interactions with microbial cells could lead to the creation of more effective antimicrobial therapies that reduce the likelihood of resistance over extended use. Future research should focus on the long-term implications of using Pt-based materials to ensure they can be safely integrated into regular antimicrobial regimens without contributing to the escalating problem of drug-resistant pathogens.

(iii) Validating anti-inflammatory applications of Pt-based active ingredients: While preliminary studies show that platinum can mitigate skin inflammation, comprehensive clinical validation is necessary to establish its efficacy and safety. Current research indicates that the anti-inflammatory effects of platinum are often enhanced when used in conjunction with photothermal therapy [205], while the direct application of platinum in skincare products without such combinations remains unexplored. This situation presents an opportunity to develop more accessible application methods by utilizing novel techniques and formulations.

(iv) Targeting sensitive skin: The effect of platinum in promoting collagen production and its antioxidant capabilities can significantly fortify skin barrier functions and mitigate the effects of external irritants. Future research should focus on combining platinum with other active ingredients to maximize its skincare benefits, especially in managing inflammation and improving cosmetic attributes. Additionally, developing targeted R&D programs for specific demographic groups could tailor therapeutic outcomes and enhance the efficacy of treatments, ensuring personalized care for sensitive skin conditions.

(v) Comprehensive safety and toxicology evaluations: The toxicological profiles of various forms of Pt-based materials, especially nanoparticles, are not fully understood, with existing research presenting conflicting results regarding their safety. This is particularly true for the long-term health implications of exposure to PtNPs, including those synthesized through bio-assisted methods. There is a need to thoroughly investigate the potential risks and benefits of these materials under different conditions, such as varying concentrations, sizes, shapes, coatings, and exposure durations. A detailed understanding of these factors is crucial for developing standardized safety guidelines and usage protocols. Additionally, comprehensive clinical trials and long-term epidemiological studies are essential to confirm the safety of these materials in dermatological applications and other consumer products, ensuring that PtNPs can be safely integrated into healthcare and cosmetic industries.

(vi) Bridging chemistry, materials science, and dermatological applications: While modern chemistry and materials science enable the production of various forms of Pt-based materials, the understanding of how synthetic changes affect their biological interactions, and, consequently, their therapeutic efficacy and safety profiles in dermatology and skincare applications, remains elusive. This is primarily due to the complex and unpredictable interactions between Pt-based materials and biological systems, which can vary significantly depending on numerous factors, including environmental and individual biological differences. Utilizing emerging technologies such as artificial intelligence [244], machine learning [245,246,247,248], and high-throughput experimentation [249], along with molecular dynamics simulations, offers promising directions for understanding how Pt-based materials interact with biological systems and how their structure and physicochemical properties contribute to successful dermatological and skincare applications. These approaches can potentially facilitate the rational design and development of Pt-based active ingredients for future dermatology and skincare applications.

## Figures and Tables

**Table 1:** A rough comparison of PtNPs synthesized by different methods.

Synthesis Methods	Reducing Agents/Stabilizers/Synthesis Conditions	Size and Shape	Dispersive State	Chemical Stability/Toxicity	Ref.
Chemical reduction	Sodium borohydride (NaBH_4_) used as a reducing agent in aqueous solution.	3.00 ± 0.50 nm, irregular	Agglomerated	Not specified	[75]
Sonoelectrochemical synthesis	2.0 mmol dm^−3^ PtCl_4_ in 0.8 mol dm^−3^ 96% ethanol, sonicated at 20 kHz and 408 kHz.	2.20 ± 0.50 nm (20 kHz), 2.30 ± 0.40 nm (408 kHz), spherical	Well-dispersed	Not specified	[75]
	A 10^−3^ M solution of chloroplatinic acid with 0.1% *w*/*w* poly(amide-hydroxyurethane) as the electrolyte, sonicated at 20 kHz with a 10% amplitude mode.	10.00–42.00 nm, spherical	Well-dispersed, capped by poly(amide-hydroxyurethane)	Not specified	[76]
Pulsed laser ablation	High-purity platinum metal sheet was ablated using a pulse laser beam (Nd: YAG laser, 532 nm wavelength, 230 mJ/pulse) in acetone, ethanol, and methanol, with energy fluxes of 25, 19, and 9 J/cm^2^, respectively.	~1.90–4.60 nm in acetone. ~2.10–4.30 nm in ethanol. ~2.30–3.30 nm in methanol. Spherical or cubic.	Agglomeration in ethanol and methanol	Acetone provides better stability (7 weeks)	[77]
	High-purity platinum metal sheet was ablated using a pulse laser beam (Nd: YAG laser, 1064 nm wavelength, 30 mJ/pulse) at repetition rates of 10 and 15 Hz.	8.00 nm at 10 Hz, 9.00 nm at 15 Hz, spherical	Agglomerated, more agglomerated at higher repetition rates	Not specified	[78]
Biosynthesis using plant extract	*Vitis vinifera* extract used as reducing agents.	1.51 ± 0.35 nm, spherical	Well-dispersed	Stable colloidal systems	[79]
	Extract of *Combretum erythrophyllum* plant leaves used as both bio-reductants and stabilizing agents.	1.04 ± 0.26 nm, spherical	Well-dispersed	−34.1 mV zeta potential, good colloidal stability	[80]
	*Phoenix dactylifera L.* fruit extract used as bio-reductants.	2.30–3.00 nm, quasi-spherical	Well-dispersed	Non-toxic in rat studies	[81]
	*Nigellasativa L.* seed extract used as reducing agents	1.00–6.00 nm, spherical	Partially aggregated	Toxic to MDA-MB-231 and HeLa cancer cells	[82]
Biosynthesis using fungi	*Neurospora crassa* fungal biomass and extracts used as reducing agents.	17.00–76.00 nm, spherical	Aggregated	Not specified	[83]
Biosynthesis using bacteria	*Acinetobacter calcoaceticus* PUCM 1011 used as reducing agents.	2.00–3.50 nm, cuboidal	Partially aggregated, embedded in organic layers	Stabilized particles	[84]

**Table 2:** Comparison between antioxidant efficiencies of various PtNPs and other metal nanoparticles.

Material	Synthesis Method	Morphology	Average Size (nm)	Concentration (µg/mL)	DPPH Scavenging Activity (%)	Ref.
PdNPs	Biosynthesized using extracts of the green alga *Botryococcus braunii*.	Spherical	4.89	25	82.27	[117]
AgNPs	Biosynthesized using purified microbial exopolysaccharides.	Spherical	30.00	50	89.50	[118]
AgNPs	Biosynthesized using an extract from the aerial parts of *Artemisia marschalliana Sprengel*.	Spherical	5.00–50.00	450	61.00	[119]
AgNPs	Biosynthesized using leaf extracts of the medicinal plant *Elephantopus scaber* L.	Spherical	50.00	250	85.90	[120]
AgNPs	Biosynthesized using aqueous extract of *Alternanthera sessilis Linn*.	Irregular shape	30.00	500	62.00	[121]
AgNPs	Biosynthesized using *Helicteres isora* root extract.	Spherical	38.23	100	90.00	[122]
AuNPs	Biosynthesized using *Nerium oleander* leaf extract.	Nearly spherical	2.00–10.00	1000	75.00	[123]
AuNPs	Biosynthesized using the aqueous extract of *P. salicifolia leaves*.	Spherical	5.00–23.00	300	57.70	[124]
AuNPs	Biosynthesized using microbial glycolipid mannosylerythritol lipid produced from *Ustilago maydis* CGMCC 5.203.	Spherical	20.33	125	55.00	[125]
AuNPs	Biosynthesized using marine bacterium *Paracoccus haeundaensis* BC74171T.	Spherical	20.93 ± 3.46	320	73.04	[107]
ZnONPs	Biosynthesized using pecan (*Carya illinoinensis*) leaf extract	Star shape	84.50	200	97.00	[126]
PtNPs	Biosynthesized using extracts of the green alga *Botryococcus braunii*.	Spherical	86.96	25	78.14	[117]
PtNPs	Biosynthesized using aqueous extract of *P. salicifolium* leaves.	Spherical	1.00–3.00	50	89.29	[116]
PtNPs	Biosynthesized using *Tragia involucrata* leaf extract.	Spherical	10.00	1000	64.00	[111]
PtNPs	Biosynthesized using red algae *Halymenia dilatata*.	Spherical	15.00 ± 1.70	100	59.72	[110]
PtNPs	Biosynthesized using *Salix Tetraspeama* Leaf extract.	Spherical	18.00 via hydrothermal-assisted synthesis; 12.00 via ultrasound-assisted synthesis	100	69.00	[108]
PtNPs	Biosynthesized using extracts of the fungus *Fusarium oxysporum*.	Cubical, spherical, and truncated triangular	28.96	25	79.14	[109]
PtNPs	Biosynthesized using acid phosphatase from *Rumex dentatus* seed extract.	Spherical	1.00–7.00	1000	88.00	[115]
PtNPs	Biosynthesized using chlorogenic acid.	Spherical	7.50–16.90 depending on the initial molar ratios of reagents	Not specified	95.00	[114]
PtNPs	Biosynthesized using *Vitis vinifera* extract	Spherical	1.51 ± 0.35	200	74.30	[79]
Pt-BSA-RSV NPs	Biosynthesized using *Enterobacter cloacae* and coated with bovine serum albumin (BSA) and resveratrol (RSV)	Irregular quasi-spherical aggregation states	222.90	400	85.00	[127]

**Table 3:** Typical personal care products using Pt-based active ingredients and their efficacy.

Product Type	Claimed Main Effects of Pt-Based Active Ingredients
Antioxidant mask	Neutralize free radicals, alleviate oxidative stress, and shield the skin from environmental pollutants and UV damage, enhancing anti-aging benefits.
Whitening serum	Regulate melanin production, lighten skin discoloration, and promote a uniform, brighter skin tone.
Anti-aging face/eye cream	Enhance collagen production, improve skin elasticity and firmness, and diminish signs of aging such as wrinkles and fine lines.
Repairing mask	Stimulate epidermal cell proliferation and collagen synthesis, accelerating skin repair.

**Table 4:** Marketed cosmetic products containing platinum and their claimed efficacy.

Brands	Series Product	Claimed Main Effects
La Prairie	Platinum Rare Haute-Rejuvenation	Contains exclusive platinum multi-peptides that reduce wrinkles and enhance skin elasticity.
Perricone MD	Cold Plasma Plus+ Platinum Trio	Contains platinum-based active ingredients that smooth wrinkles, firm skin, and visibly correct discoloration and redness.
Elizabeth Grant	Collagen Re-Inforce Platinum 24 Hour Firming Creme	Contains platinum-based active ingredients that promote collagen production, providing all-day firming and moisturizing.
Cada Suissesse	Stem Cellue Nano Platinum Skin Rejuvenating Cream	Utilizes platinum nanoconjugates to rejuvenate aging skin, improve elasticity, and diminish wrinkles.
Skin Advanced	Platinum Liposome	Utilizes non-nano-sized platinum liposomes to boost antioxidant levels, hydrate, strengthen the skin barrier, and reduce redness.

Note: The claimed effects are based on product descriptions extracted from the official website of each brand.

## Data Availability

Not applicable.

## Abbreviations

5,6-DHI	5,6-Dihydroxyindole
5,6-DHICA	5,6-dihydroxyindole-2-carboxylic acid
ACP-PtNPs	Platinum nanoparticles synthesized by acid phosphatase
ANOVA	Analysis of variance
AgNPs	Silver nanoparticles
ATP	Adenosine triphosphate
Au	Gold
AuNPs	Gold nanoparticles
AuPtNPs	Gold and platinum bimetallic nanoparticles
CAT	Catalase
COX-2	Cyclooxygenase-2
CP-Au/Pt	Citrate- and pectin-protected gold-platinum bimetallic nanoparticles
CP-Pt	Citrate- and pectin-protected platinum nanoparticles
DPPH	2,2-diphenyl-1-picrylhydrazyl
DNA	Deoxyribonucleic acid
DSPE-PEG	1,2-distearoyl-sn-glycero-3-phosphoethanolamine-polyethylene glycol
ERK	Extracellular signal-regulated kinase
GPx	Glutathione peroxidase
GSH	Glutathione
HepG2	Human hepatocellular carcinomas
HLB	Hydrophilic–lipophilic balance
H_2_PtCl_6_	Chloroplatinic acid
IFN-γ	Interferon-γ
IMQ	Imiquimod
iNOS	Inducible nitric oxide synthase
JNK	c-Jun NH (2)-terminal kinase
LPtNP	Large platinum nanoparticle
L1-PtNPs	Platinum nanoparticles synthesized with a 1:1 ratio of chloroplatinic acid to *N. tetragona*
L4-PtNPs	Platinum nanoparticles synthesized with a 1:4 ratio of chloroplatinic acid to *N. tetragona*
MAPKs	Mitogen-activated protein kinases
MNs	Microneedles
NF-κB	Nuclear factor kappa-light-chain-enhancer of activated B cells
NQO1	NAD(P)H quinone dehydrogenase 1
O_2_^−^	Superoxide anions
OH^−^	Hydroxyl
Pd	Palladium
PdNPs	Palladium nanoparticles
PEG	Polyethylene glycol
PGEs	Platinum group elements
PLGA	Poly(d,l-lactic-co-glycolic acid)
POD	Peroxidase
Pt	Platinum
Pt (0)	Platinum (0)
Pt (IV)	Platinum (IV)
Pt-apo	Platinum nanoparticles encased in apoferritin protein
Pt-BSA-RSV NPs	Platinum nanoparticles coated with bovine serum albumin and resveratrol
PtNPs	Platinum nanoparticles
PtNWs	Platinum nanowires
PtNZ	Platinum-based nanozyme
RhNPs	Rhodium nanoparticles
RNA	Ribonucleic acid
ROS	Reactive oxygen species
SOD	Superoxide dismutase
SPtNP	Small platinum nanoparticle
TGF-β	Transforming growth factor-beta
TOAB	Tetra-n-octylammonium bromide
UV	Ultraviolet
UVA	Ultraviolet A
UVB	Ultraviolet B
ZnONPs	Zinc oxide nanoparticles
